# Global, regional and national availability of essential medicines for children, 2009–2020: a systematic review and meta-analysis

**DOI:** 10.1186/s12889-023-15820-7

**Published:** 2023-06-20

**Authors:** Yuqing Shi, Zhe Chen, Kun Zou, Miao Zhang, Zheng Liu, Dan Liu, Linan Zeng, Hailong Li, Zhi-Jun Jia, Guo Cheng, Yong Tang, Shaoyang Zhao, Yongmu Jiang, Imti Choonara, Lingli Zhang

**Affiliations:** 1 Department of Pharmacy, West China Second University Hospital, Sichuan University, Chengdu, China; 2 Evidence-Based Pharmacy Center, West China Second University Hospital, Sichuan University, Chengdu, China; 3 NMPA Key Laboratory for Technical Research on Drug Products In Vitro and In Vivo Correlation, Chengdu, China; 4 Key Laboratory of Birth Defects and Related Diseases of Women and Children, Sichuan University, Ministry of Education, Chengdu, China; 5 West China School of Pharmacy, Sichuan University, Chengdu, China; 6 West China School of Medicine, Sichuan University, Chengdu, China; 7 Department of Pediatrics, West China Second University Hospital, Sichuan University, Chengdu, China; 8 Laboratory of Molecular Translational Medicine, Center for Translational Medicine, Sichuan University, Chengdu, China; 9 School of Economics, Sichuan University, Chengdu, China; 10 School of Medicine, University of Nottingham, Nottingham, DE22 3DT UK; 11 Chinese Evidence-based Medicine Center, West China Hospital, Sichuan University, Chengdu, China

**Keywords:** Children, Essential medicine, Availability, Systematic review

## Abstract

**Background:**

Access to essential medicines is a vital component of universal health coverage. The low availability of essential medicines for children (EMC) has led the World Health Organization (WHO) to issue a number of resolutions calling on member states on its improvement. But its global progress has been unclear. We aimed to systematically evaluate the progress of availability of EMC over the past decade across economic regions and countries.

**Methods:**

We searched eight databases from inception to December 2021 and reference lists to identify included studies. Two reviewers independently conducted literature screening, data extraction and quality evaluation. This study was registered with PROSPERO, CRD42022314003.

**Results:**

Overall, 22 cross-sectional studies covering 17 countries, 4 income groups were included. Globally, the average availability rates of EMC were 39.0% (95%CI: 35.5-42.5%) in 2009–2015 and 43.1% (95%CI: 40.1-46.2%) in 2016–2020. Based on the World Bank classification of economic regions, income was not proportional to availability. Nationally, the availability rate of EMC was reasonable and high (> 50%) in only 4 countries, and low or very low for the rest 13 countries. The availability rates of EMC in primary healthcare centers had increased, while that for other levels of hospitals slightly declined. The availability of original medicines decreased while that of generic medicines was stable. All drug categories had not achieved the high availability rate.

**Conclusion:**

The availability rate of EMC was low globally, with slight increase in the last decade. Continuous monitoring and timely reporting of the availability of EMC are also needed to facilitate targets setting and inform relevant policy making.

**Supplementary Information:**

The online version contains supplementary material available at 10.1186/s12889-023-15820-7.

## Background

Access to essential medicines is a vital component to the fulfilment of the right to the highest attainable standard of health [1]. As one of the Sustainable Development Goals of the United Nations, access to safe, effective, quality, and affordable essential medicines is important to health coverage for children by 2030 [2]. In the last ten years, to improve availability of essential medicines for children (EMC), the World Health Organization (WHO) has issued some resolutions calling on member states to focus on it and also has regularly updated the model list of EMC. [3–5] The first Essential Medicines List for Children was published in 2007. The current versions, updated in September 2021, are the 8th Essential Medicines List for Children (EMLc). [6].

To promote and standardize investigation on the availability, price, and affordability of essential medicines, WHO and Health Action International (HAI) have developed “Measuring medicine prices, availability, affordability and price components” in 2003. [5] Previous studies found that the availability and affordability of EMC was generally low in low- and middle-income countries (LMCs). [7,8] However, it is unclear whether there is a progress on the availability of EMC over the last decade globally, to what extent, and its variations among countries. Therefore, this systematic review and meta-analysis was conducted to examine the global trend on the availability of EMC, its variation among economic regions and countries, to provide benchmarking and inform evidence-based health policy-making.

## Methods

### Search strategy and selection criteria

We conducted a systematic review in accordance with the Preferred Reporting Items for Systematic Reviews and Meta-Analyses (PRISMA) guidelines. [9] The protocol of this systematic review was registered on PROSPERO, CRD42022314003.

We systematically searched PubMed, Embase, the Cochrane Library, Web of Science, CNKI, China Biology Medicine (CBM) from inception to December 2021. We also searched websites of WHO and Health Action International, reference lists of included studies, and published reviews for more eligible studies. The search strategy included a combination of medical subject headings and free text terms for (“child*” or “pediatri*”) and (“essential medic*” or “essential drug*”) and was adapted for each database.

The studies were included if they investigated medical institutions or pharmacies for availability of EMC, including vaccines, and they were designed as cross-sectional study. The studies published in all languages were included. Studies were excluded if they were: editorials, conference abstracts, and any unobtainable full-texts. Two reviewers (SYQ and CZ) selected studies according to the inclusion and exclusion criteria independently. Discrepancies were resolved by discussion or consultation with a third reviewer.

### Data extraction and analysis

Two reviewers (SYQ and CZ) independently extracted data using predefined excel data extraction form. The extracted data included the first author, published year, survey area, survey time, methods, the availability of EMC. Joanna Briggs Institute (JBI) was used to evaluate the risk of bias of included studies. [10] This tool has nine items and categorized each item with “yes”, “unclear” and “no”, and gave them “1” and “0” points. The total score > 6 points was considered as high quality, between 4 and 6 points was considered as medium quality; < 4 was considered as low quality. [11].

### Statistical analysis

The availability rate was defined as the percentage of facilities with stock of the required on the day survey data were collected. We extracted the number of the facilities that had the medicine and total number of facilities on the day of data collection. Data were meta-analyzed using Stata 15.1 (Stata Corp, College Station, TX). The final availability rates were presented as weighted average and their 95% confidence intervals (CIs).

They were estimated by world, economic levels (The World Bank groups - high-income, upper-middle-income, lower-middle-income and low-income countries), country, types of medical institutions (ownership - public and private; institutional level - primary healthcare and other levels hospital), and medicine classification using the Anatomical Therapeutic Chemical (ATC) code. We also analyzed results across the six WHO geographical regions, but were excluded due to the small number of studies and lack of representativeness.

The United Nations Millennium Development Goals (MDGs) are goals that UN Member States have agreed to try to achieve by the year 2015. Therefore, we estimated the availability rate for 2009–2015 years and 2016–2020 years, respectively, to quantify its progress over time. [2].

The availability rate of essential medicines was classified as: not available (availability = 0); very low (0 < availability < 30%), low (30%≤availability < 50%), reasonable (50%≤availability < 80%) and high (≥ 80%) [12]. *I*^2^ test and Chi square test were used to examine heterogeneity. *I*^*2*^ > 50% or *P* ≤ 0.05 indicated significant heterogeneity among studies, where random effect model was used. Otherwise, fixed effect model was used.

## Results

### Study characteristics

**Fig. 1:**
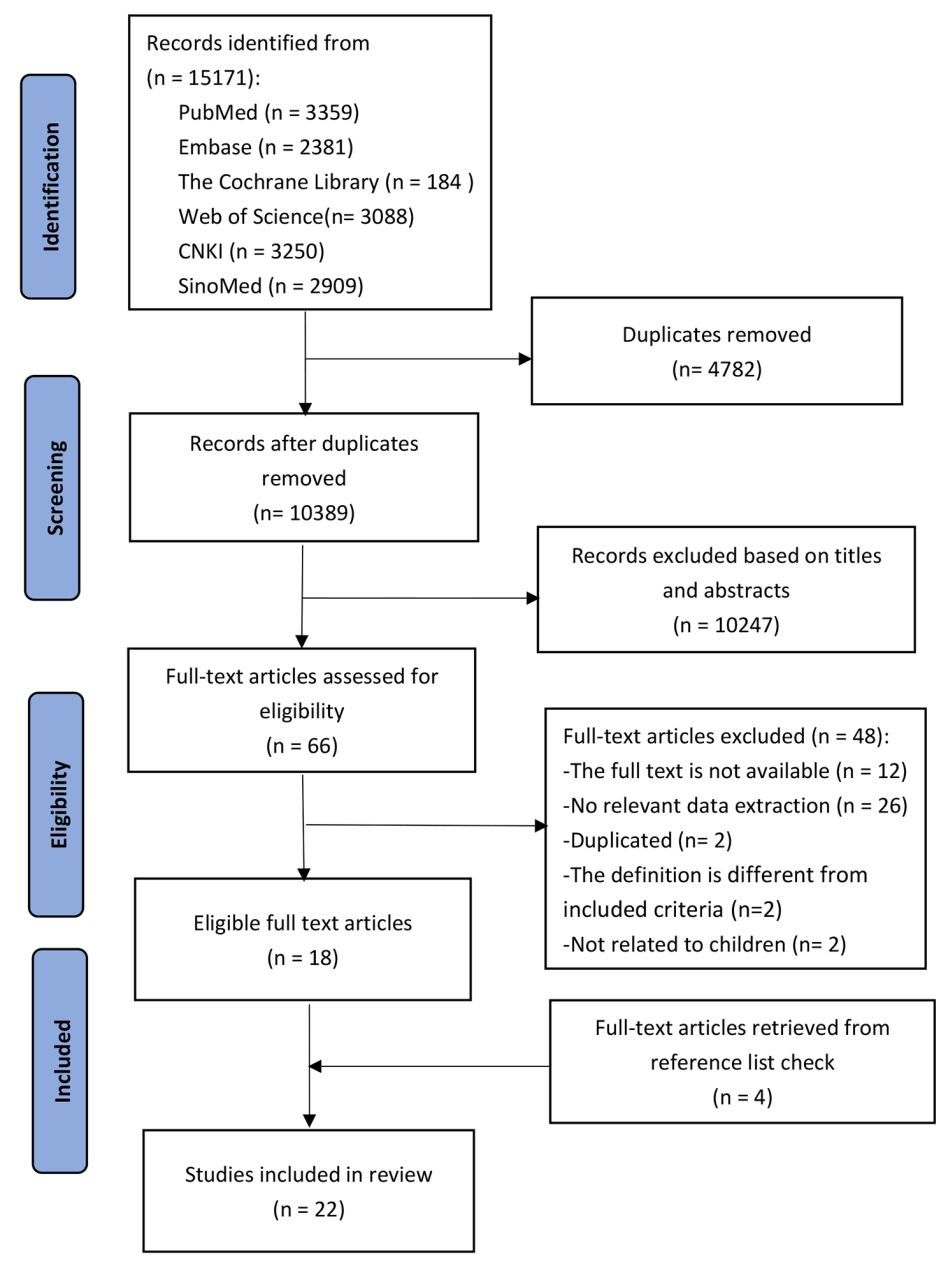
PRISMA flowchart of study selection

We identified 15,171 unique titles for eligibility screening, and 22 studies were finally included after assessment of the full-text manuscript by two reviewers independently. (Fig. 1) The characteristics of the included studies were summarized in Table 1. All studies were cross-sectional studies conducted from 2009 to 2020. All included studies were from low-income (3/22), lower-middle-income (12/22), upper-middle-income groups (6/22), and one study (1/22) involved 8 LICs, 16 LMCs, 15 UMCs and 19 HICs. Methodologically, 15 (68.2%) studies used standardized WHO/HAI methodology, [14, 16–18, 20, 22–26, 30–34] 2 (9.1%) studies used adapted WHO/HAI method, [13, 29] and the remaining 5 (22.7%) studies used other methods. [15,19, 21, 27, 28] The mean number of essential medicines investigated was 31 (range: 5 to 121).

**Table 1:** Descriptive characteristics of included studies

First author, year	Methods	Survey time	Country	World Bank country income groups	Number of essential medicines	Investigated EMC	Mean availability rate		
							Public Sector		Private Sector
Balasubramaniam, 2011 [13]	Adapted WHO/HAI	2009	Sri Lanka	LMC	25	/	52.0%		80.0%
Balasubramaniam, 2014 [14]	WHO/HAI	2009	Sri Lanka	LMC	25	/	/		
Gitanjali, 2011 [15]	Snapshot survey	2010	India	LMC	5	NRHM	80.0%		/
Anson, 2012 [16]	WHO/HAI	2010	Guatemala	LMC	22	WHO EMLc	46.0%		35.0%
Swain, 2015 [17]	WHO/HAI	2010	India	LMC	34	Childspecific essential medicines list for the state	**LPMs**: 17.0%		**LPMs**: 38.5%
							**HPMs**: 0.0%		**HPMs**: 10.8%
Wang, 2014 [18]	WHO/HAI	2012	China	UMC	28	WHO EMLc: n = 21 Other list ^1^: n = 7	**OBs**: 10.8%		**OBs**: 11.9%
							**LPGs**: 27.3%		**LPGs**: 20.6%
Droti, 2019 [19]	SARA survey	2012	Benin	LIC	12	Priority life-saving medicines for women and children	50.0%		
			Burkina Faso				54.0%		
			Democratic Republic of the Congo				28.0%		
			Mauritania				30.0%		
			Sierra Leone				50.0%		
			Togo				57.0%		
			Uganda				44.0%		
			Zimbabwe				54.0%		
Wang Xiao, 2014 [20]	WHO/HAI	2013	China	LMC	28	WHO EMLc: n = 21 Other list ^5^: n = 7	/	**OBs**: 11.9%	
								**LPGs**: 20.6%	
Pujari, 2016 [21]	Simple direct-contact methodology	2015	India	LMC	10	NRHM and WHO EMLc	**HPMs**: 0.00%	**HPMs**: 10.8%	
							**LPMs**: 17.0%	**LPMs**: 38.5%	
Sado, 2016 [22]	WHO/HAI	2015	Ethiopia	LIC	22	WHO EMLc	**HPMs**: 1.2%	**HPMs**: 7.4%	
							**LPMs**: 43.0%	**LPMs**: 42.8%	
Dorj, 2018 [23]	WHO/HAI	2016	Mongolia	LMC	30	WHO EMLc	72.6%	76.7%	
Li, 2018 [24]	WHO/HAI	2016	China	UMC	121	WHO EMLc/EML in China: n = 87 EML in China: n = 34	**14 WHO EMLc medicines**: 21.3%		
Orubu, 2019 [25]	WHO/HAI	2016	Nigeria	LMC	12	WHO EMLc	/		
Sun, 2018 [26]	WHO/HAI	2017	China	UMC	40	WHO EMLc: n = 29 Other list ^2^: n = 11	**OBs**: 7.5%	**OBs**: 8.9%	
							**LPGs**: 34.2%	**LPGs**: 29.4%	
Martei, 2020 [27]	Questionnaire survey	2017	/	HIC, UMC, LMC, LIC	29	WHO EMLc: n = 3 SIOP core medicines: n = 18 SIOP ancillary list: n = 8	/		
Dai, 2020 [28]	Current situation survey method	2017	China	UMC	42	WHO EMLc: n = 32 EML in China: n = 10	**OBs**: 33% **Generics**: 32%		
Faruqui, 2019 [29]	Adapted WHO/HAI	2018	India	LMC	28	WHO EMLc	**Non-cancer EMs**: 69.0%	**Non-cancer EMs**: 58.0%	
							**Anti-neoplastic EMs**: 43.0%	**Anti-neoplastic EMs**: 71.0%	
Wei, 2019 [30]	WHO/HAI	2019	China	UMC	49	WHO EMLc EML in China WHO-HAIM list	**Year 2012**: 41.15% **Year 2016**: 30.99%		
Wang, 2020 [31]	WHO/HAI	2019	China	UMC	30	WHO EMLc: n = 24 Regional Supplemental list: n = 6	**OBs**: 15.8%	**OBs**: 30.3%	
							**LPGs**: 44.8%	**LPGs**: 36.7%	
Tadesse, 2021 [32]	WHO/HAI	2019	Ethiopia	LIC	30	WHO EMLc: n = 23 Other list ^4^: n = 7	/	**HPMs**: 3.87%	
							**LPMs**: 57.67%	**LPMs**: 53.67%	
Dinh, 2021 [33]	WHO/HAI	2020	Viet Nam	LMC	30	WHO EMLc: n = 14 Other list ^3^: n = 16	**HPMs**: 5.8%	**HPMs**: 11.3%	
							**LPMs**: 24.9%	**LPMs**: 33.2%	
Mensah, 2021 [34]	WHO/HAI	2020	Ghana	LMC	28	WHO EMLc	**Non-cancer EMs**:38.0%	**Non-cancer EMs**: 84.0%	
							**Anti-neoplastic EMs** 27.0%	**Anti-neoplastic EMs**:75.0%	

***Note***: ^1^*The 2009 NEML, Shaanxi Provincial Essential Medicine Supplementary List and the opinions of several groups of experts;*

^2^ *The 11 medicines were identified as supplementary medicines, which were selected based on the local children’s disease needs, the 2012 NEML, feedback from several pediatric experts and literature reviews;*

^3^ *The 9 medicines from WHO’s core list not registered in Vietnam were removed and superseded by nine supplementary medicines (similar active ingredients, but alternate dosage forms and/or strengths). 7 supplementary medicines were chosen to treat important national health problems and the burden of local diseases for children (such as pneumonia, diarrhoea, colds and pain);*

^4^ *7 medicines were added to the study list as per the prevalence and burden of diseases associated with childhood illness in the region (SNNP Regional Health bureau);*

^5^ *Regional Supplemental Directory*

*WHO EMLc, the WHO Model Lists of EMC; Snapshot survey, a simple survey form prepared through Google documents was used to collect data; NRHM, the National Rural Health Mission’s list; SIOP, International Society of Pediatric Oncology; SARA Survey, data are collected using WHO’s standard SARA core questionnaire, with adaptations to country context for facility classifications, subnational administrative units and staff categories. HIC, high-income country; UMC, upper-middle income country; LMC, lower-middle-income country; LIC, low- income country; OBs, original brands; LPGs, lowest priced generics; LPMs, lowest priced medicines; HPMs, highest priced medicines; EMs, essential medicines*

### Risk of bias assessment

Seventeen studies (77.3%) selected surveyed essential medicines according to the WHO Model List of EMC (EMLc), [16, 18, 20–34] while other studies selected surveyed essential medicines from other sources (including “priority life-saving medicines for women and children” developed by the WHO, list of national rural health mission, childspecific essential medicines list of the state, international society of pediatric oncology, and regional supplemental directory). The source of the list in 2 studies was unknown. [13, 14]

Most studies (21, 95.5%) were rated as low risk of bias, [13–27, 29–34] whereas one study (4.5%) was graded as medium risk. [28] 11 studies achieved 9 points and 9 studies scored 8 points. There was one article each for 6 and 7. The bias was mainly from the following items: five studies (22.8%) were considered as unwell-represented samples. [14, 15, 24, 27, 30] Four studies (18.2%) were considered as insufficient coverage of the identified sample. [15, 19, 23, 24] It is unclear that if the sample size was adequate in nineteen studies (86.4%),^13–19, 21–[24, 26–31, 33]–[34]^ and coverage of the identified sample was sufficient in eleven studies (50.0%). ^[14, 16–18, 21, 22, 25–27, 29], 31^The criteria and results of quality assessment for each study were shown in **Supplementary Table **2.

### Global trend of availability of essential medicines for children

**Fig. 2:**
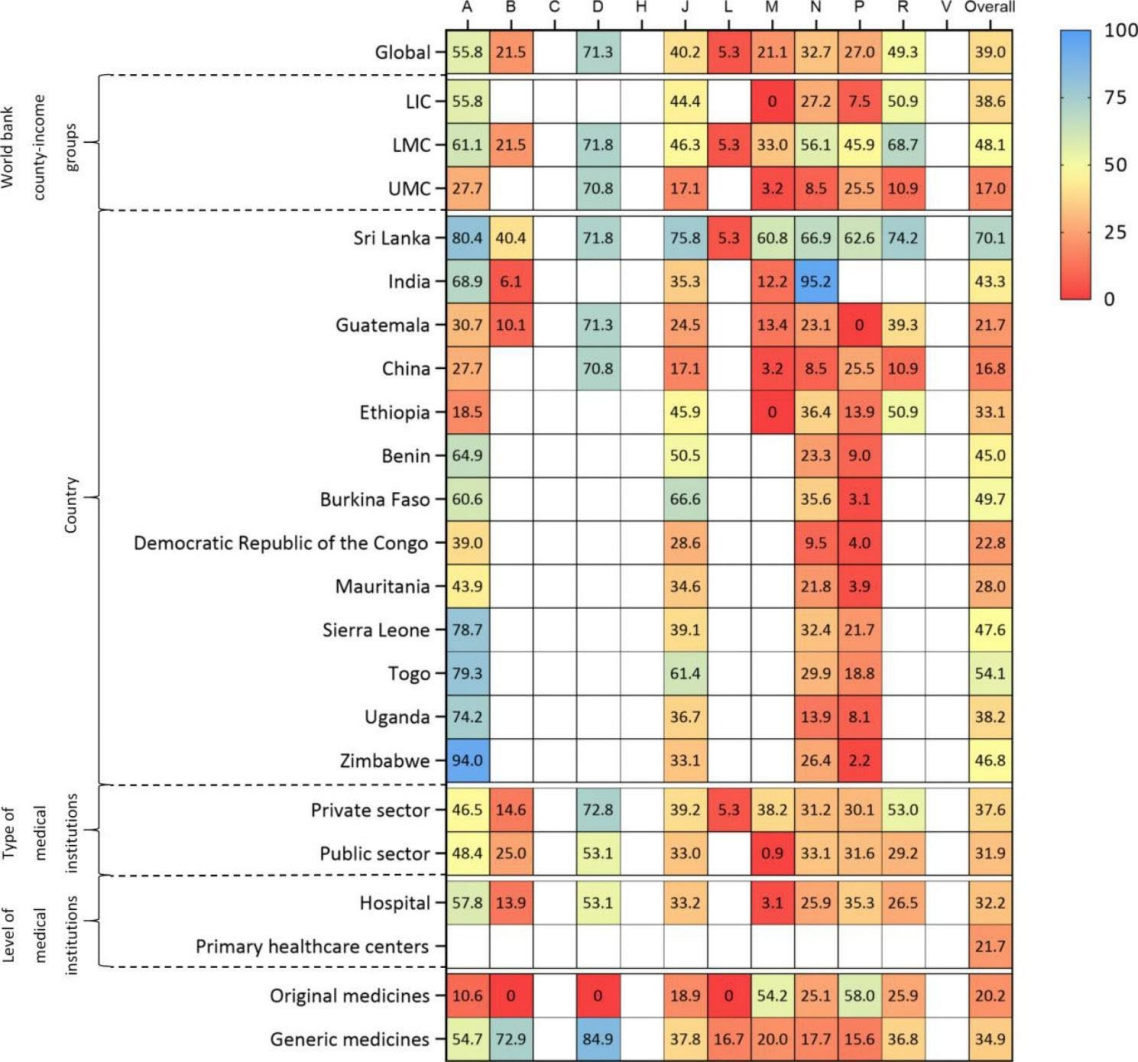
Availability rates (%) of essential medicines for children from 2009–2015 ***Note***: A, Alimentary Tract And Metabolism; B, Blood And Blood Forming Organs; C, Cardiovascular System; D, Dermatologicals; G, Genito Urinary System And Sex Hormones; H, Systemic Hormonal Preparations, Excl. Sex Hormones And insulin; J, Antiinfectives For Systemic Use; L, Antineoplastic And Immunomodulating Agents; M, Musculo-Skeletal System; N, Nervous System; P, Antiparasitic Products, Insecticides And Repellents; R, Respiratory System; V, Various

**Fig. 3:**
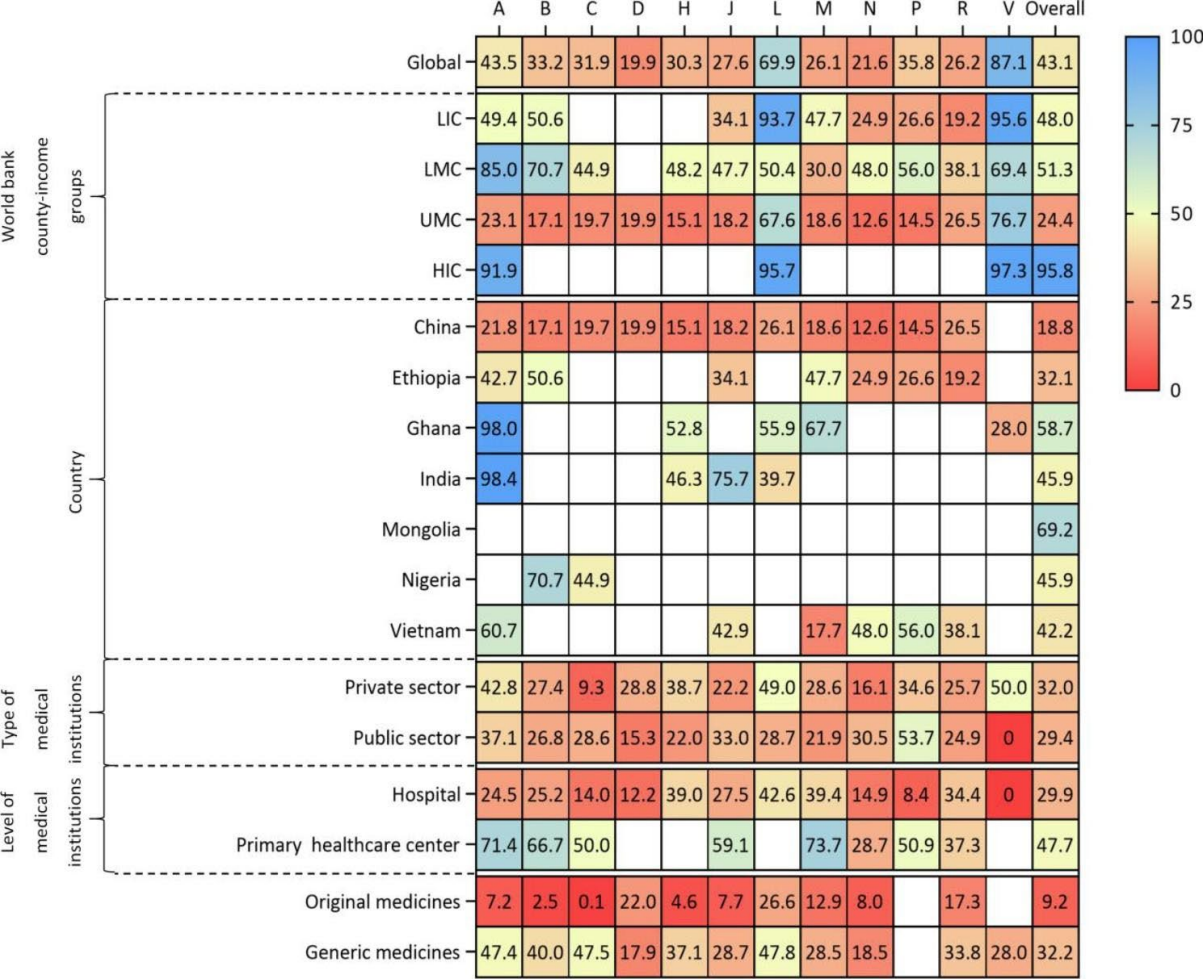
Availability rates (%) of essential medicines for children from 2016–2020 ***Note***: A, Alimentary Tract And Metabolism; B, Blood And Blood Forming Organs; C, Cardiovascular System; D, Dermatologicals; G, Genito Urinary System And Sex Hormones; H, Systemic Hormonal Preparations, Excl. Sex Hormones And insulin; J, Antiinfectives For Systemic Use; L, Antineoplastic And Immunomodulating Agents; M, Musculo-Skeletal System; N, Nervous System; P, Antiparasitic Products, Insecticides And Repellents; R, Respiratory System; V, Various

Overall, the availability rate of EMC from 2009 to 2015 was 39.0% (95%CI: 35.5-42.5%), while that from 2016 to 2020 was 43.1% (95%CI: 40.1-46.2%). There was a high degree of heterogeneity between the studies in 2009–2015 and 2016–2020 (*I*^*2*^ = 98.44%, *I*^*2*^ = 94.23%), so the random effect model was used. (Figs. 2 and 3)

### Economic regional availability of essential medicines by world bank country-income groups

**Fig. 4:**
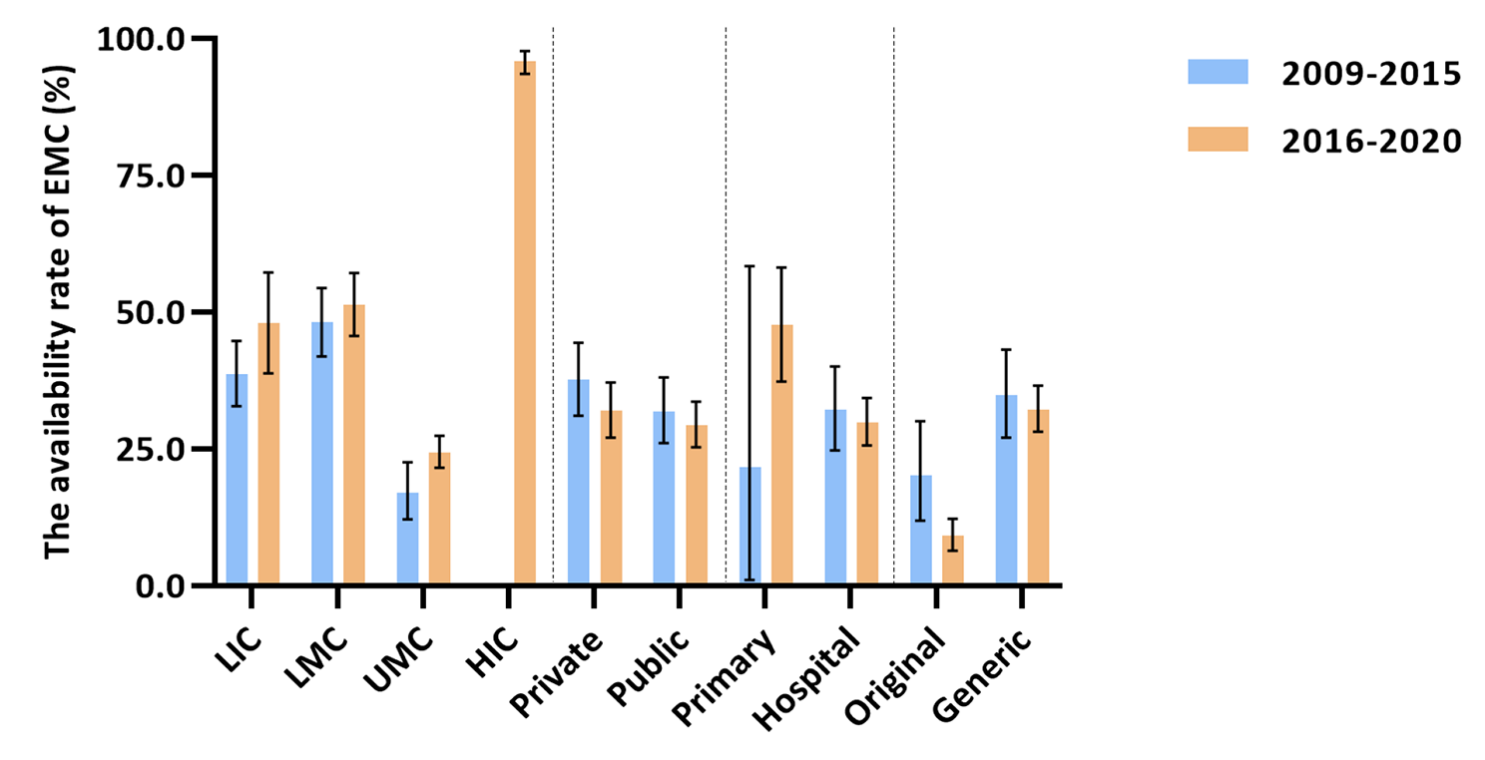
Availability rates of essential medicines for children by income, ownership, level of medical institutions, original and generic medicines, 2009 to 2015 and 2016 to 2020 ***Note***: HIC, high-income country; UMC, upper-middle income country; LMC, lower-middle-income country; LIC, low- income country; Private, private medical institutions; Public, public medical institutions; Primary, primary healthcare centers; Hospital, other levels of hospitals except for primary medical institutions; Original, original medicines; Generic, generic medicines

By World Bank classification of income countries, the availability rate of EMC in LMC countries was the highest (48.1%, 95%CI: 41.9-54.4%) from 2009 to 2015. (Fig. 2) From 2016 to 2020, the availability rate of EMC was highest in HIC countries (95.8%, 95%CI: 93.5-97.7%). (Figs. 3 and 4)

**Fig. 5:**
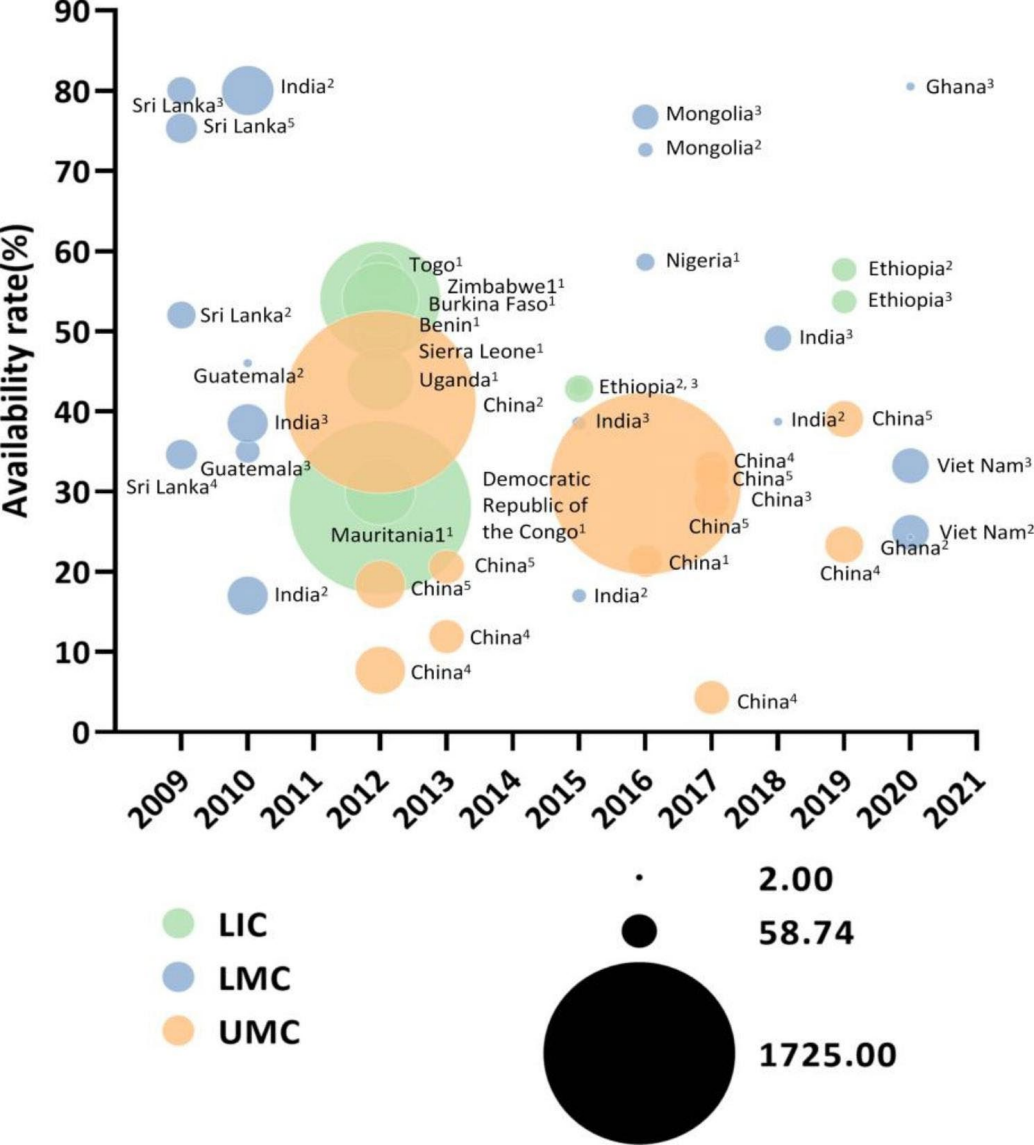
Changes of availability rates (%) of essential medicines for children over time from 2009–2020 ***Note***: LIC, low- income country; LMC, lower-middle-income country; UMC, upper-middle income country. ^1^ Total availability; ^2^ Public sectors; ^3^ Private sectors; ^4^ Original medicines; ^5^ Generic medicines. The size of the dots indicate the number of surveyed institutions

As was shown in the Fig. 5, there was no proportional between income and availability rates, and the regularity of changes between time and availability rates was not obvious.

### Availability of essential medicines for children by countries

**Fig. 6:**
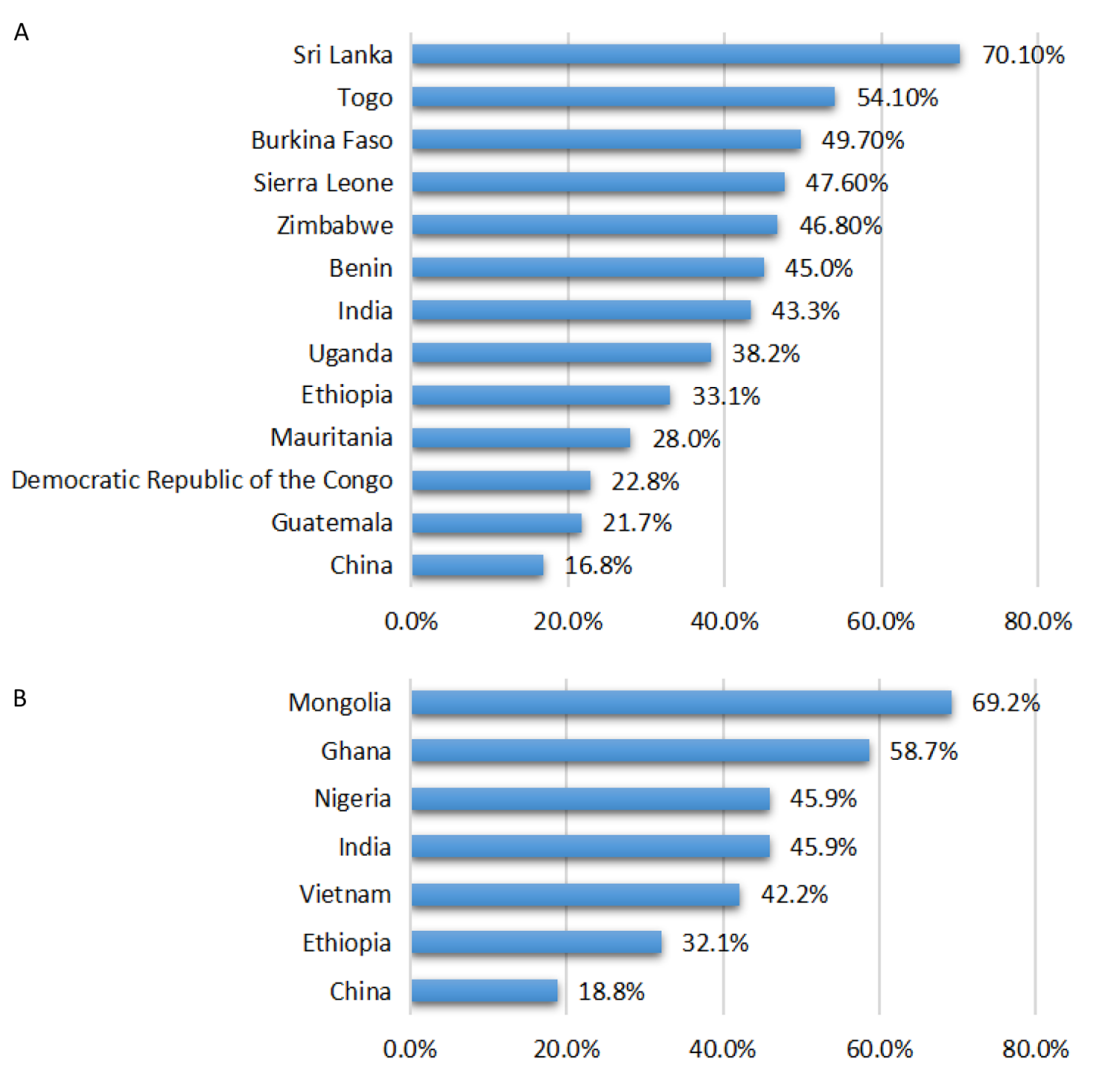
Availability rates of essential medicine for children by country, from 2009–2015 **(A)** and from 2016–2020 **(B)** *Note: The availability rate of medicines was calculated as percentage (%) of the surveyed outlets where the medicines were found on the day of data collection.* The values in the bar chart were pooled average availability rates of all surveyed medicines in each country using Stata 15.1.

Only 13 and 7 countries reported country-specific availability rate of EMC from 2009 to 2015 and from 2016 to 2020, respectively. From 2009 to 2015, the availability rate of EMC ranged from 16.8% (95% CI: 12.0%, 22.2%) in China to 70.1% (95% CI: 62.0-77.6%) in Sri Lanka. (Fig. 2) From 2016 to 2020, the availability rate of EMC was lowest in China (18.8%, 95%CI: 16.2-21.5%) and highest in Mongolia (69.2%, 95%CI: 58.6-79.0%). (Fig. 3) There were studies conducted in India, China and Ethiopia in both of the two periods, and the availability rates of India and China increased by 2.6% and 2.0%, respectively. While the availability rates of Ethiopia decreased by 1.0%. (Fig. 6)

### Availability of essential medicines for children by types of medical institutions

By ownership of medical institutions, the availability rate of EMC was 31.9% (95% CI: 26.0-38.0%) in public medical institutions, lower than that of 37.6% (95% CI: 31.0-44.4%) in private medical institutions from 2009 to 2015. (Fig. 2) From 2016 to 2020, the availability rate of EMC in public medical institutions was 29.4% (95% CI: 25.3-33.6%), still lower than that of 32.0% (95% CI: 27.0-37.1%) in private medical institutions. (Figs. 3 and 4)

By level of medical institutions, the availability rate of EMC was 21.7% (95% CI: 1.0-58.4%) in primary healthcare centers, lower than that of 32.2% (95% CI: 24.7-40.0%) in higher level hospitals from 2009 to 2015. (Fig. 2) From 2016 to 2020, the availability rate of EMC in primary healthcare centers increased by 26.0%, while that of other level hospitals decreased by 2.3%. (Figs. 3 and 4)

### Availability of original and generic essential medicines for children

The availability rate of generic medicines was much higher than original medicines. From 2009 to 2015, the availability rate of generic medicines was 34.9% (95% CI :27.0-43.1%), while that of original medicines was 20.2% (95% CI:11.9-30.0%). (Fig. 2) From 2016 to 2019, the availability rate of generic medicines was 32.2% (95% CI: 28.1-36.5%) and that of original medicines was 9.2% (95% CI: 6.4-12.2%), showing an enlarged gap between generic medicines and original medicines. (Figs. 3 and 4)

### Availability of medicine categories according to ATC

**Fig. 7:**
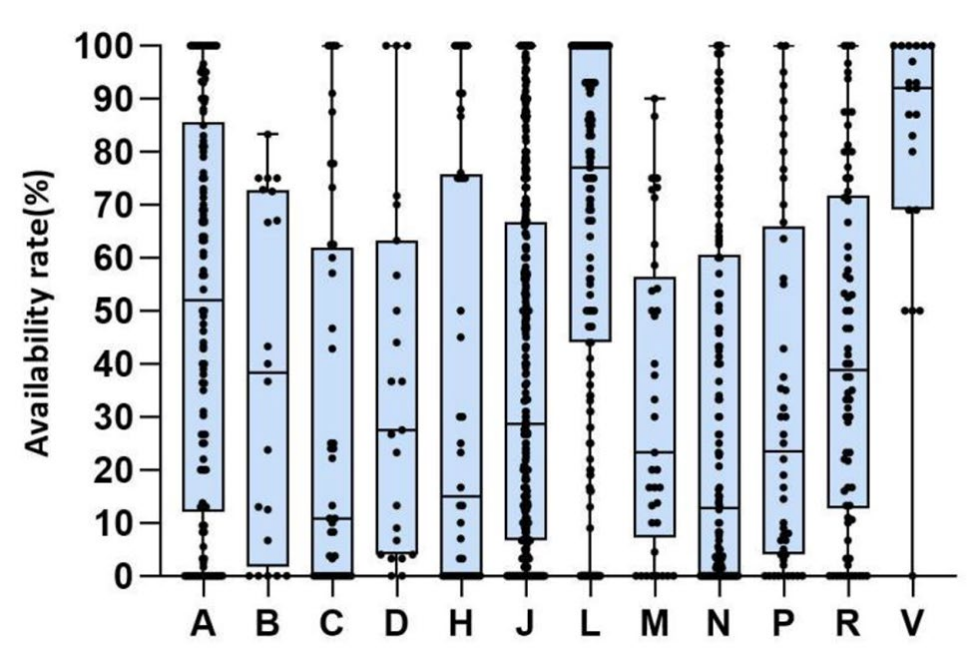
Availability of essential medicines for children by ATC, 2009 to 2020 ***Note***: A, Alimentary Tract And Metabolism; B, Blood And Blood Forming Organs; C, Cardiovascular System; D, Dermatologicals; J, Antiinfectives For Systemic Use; L, Antineoplastic And Immunomodulating Agents; M, Musculo-Skeletal System; N, Nervous System; P, Antiparasitic Products, Insecticides And Repellents; R, Respiratory System; V, Various Box limits indicate the range of the central 50% of the data, with a central line marking the median value. Lines extend from each box to capture the range of the remaining data. The black dots represent each specific availability value of ATC.

The results showed that there were significant differences in the availability rate of different categories of medicines. From 2009 to 2020, 5 categories were scored as very low (C, N, M, H, P), low for 5 categories (B, J, D, R, A), reasonable for 1 category (L) and high for 1 category (V). Cardiovascular System had lowest availability (24.1%, 95%CI: 13.6-36.2%) and Antineoplastic and Immunomodulating Agents had highest availability (69.2%, 95%CI: 64.0-74.3%). (Fig. 7)

## Discussion

In this study, we comprehensively assessed the global availability of EMC from 2009 to 2020. The global availability rate of EMC in 2016–2020 (43.1%) increased, but not significantly compared with that in 2009–2015 (39.0%). By World Bank country income groups, income was not proportional to availability. By country, the availability rate varied substantially across countries in both 2009–2015 and 2016–2020. By ownership of medical institutions, the availability rate of EMC in public medical institutions was slightly lower than that of in private medical institutions in both 2009–2015 and 2016–2020. By level of medical institutions, the availability rates in primary healthcare centers had increased, while that for higher level hospitals had slightly declined. In terms of generic and original medicines, the availability of generic medicines was stable but that of original medicines decreased. The availability rate of EMC also varied largely for drug categories, from low to reasonable availability. Furthermore, our findings showed that access to essential medicines for major noncommunicable diseases hadn’t reached the target of 80% [35].

As far as we know, this study is the first to quantify the availability rate of EMC and its trend with time globally, nationally and regionally for the last decade. The global availability rate of EMC had slightly increased in the last decade. There was substantial variability between countries and economic regions. However, there was a relatively limited number of economic regions and countries involved in surveys, indicating substantial research gaps globally, regionally and nationally. Caution is also needed in generalizing the availability rate of a country as representative of the whole economic region. We recommend that countries continue to conduct surveys on the availability of EMC to fill the current lack of data.

In terms of relationship of availability and income, there are several possible reasons for lower levels of EMCs in UMCs or than LMCs. Firstly, LMCs may focus on essential medicines from the WHO EMLc (covered by the survey); that UMCs often use other branded/combination medicines not on the EML, so their score were low on WHO EMLc medicines. Besides, an article surveyed availability of essential cytotoxic medicines for treating children with cancers in 50 countries also concluded that income is not proportional to availability, and analyzed that was attributed to a narrower range of clinical protocols or manage lower stages of disease and therefore rely on a smaller number of agents in LICs. [27] Finally, national policies may facilitate access to medicines. Sri Lanka, though a lower-middle income country, had a high availability rate, which might be related to its national policies. It had established a national pharmaceutical company to distribute drugs to the public and private sectors, and the public sector provided free essential medicines to residents. [13] China, a upper-middle income country, had officially implemented the essential medicine system since 2009, but there was a low availability of EMC, due to the high cost of development and low pricing, leading to lose motivation for enterprises. Secondly, the implementation of the policy of China’s list of essential medicine for children has been slow. Thirdly, the varieties of essential medicines procured by medical institutions at all levels are not fully consistent with EMLc. Therefore, China also needs to further implement the policy, formulate incentive measures, and encourage enterprise to invest. [24] In addition, there are other countries that increased availability rate through reforms. Uganda had improved access to cancer medicines from 28.5 to 85.8% within a space of 2 fiscal years by two main factors: first, redefining cancer medicines as highly specialized drugs and legalizing an independent procurement in 2016; second, by streamlining the procurement and supply chain to eliminate or minimize the role of middlemen. [36] The Mexico government had implemented pooled procurement to improve availability. [37].

We also found that the availability rate of EMC in public medical institutions was slightly lower than that of in private medical institutions in both 2009–2015 and 2016–2020, which were similar to a systematic review of essential medicines for asthma. [38] While private sector is important to facilitate access to EMC, more measures are warranted to increase the availability of EMC in public health institutions, for they are the main providers of public health and primary healthcare services and pivotal in pursuing universal health coverage in many countries.

The gap between the availability of original and generic medicines is growing. On the one hand, countries encourage the research and development of generic medicines, promoting higher availability of generic medicines. On the other hand, they also need to encourage the research and development of original medicines, establish an early reliable patent warning system of medicines, and protect intellectual property rights. [39] Therefore, introducing incentives for production infrastructure and suppliers improvements is crucial, including financial incentives and policy support to address the economic causes of manufacturing issues. [40].

There is also a large variation of availability rates among medicine categories, ranging from 24.1 to 87.1% in 2009–2020. Among them, the anti-neoplastic and immunomodulating agents had the highest availability rate. Among the survey institutions with an availability rate of > 80%, the type of institution and the level of the medical institution were not clearly indicated in part of studies due to the small number of available studies. Therefore, it could not be explained from this perspective. Analyzing income levels > 80% of the countries, high-income and upper-middle-income countries accounted for more than half, similar to the findings of a global cross-sectional survey of 82 countries. [41] Although availability was also high in low-income countries, the data are all from one study with only eight institutions, making them unrepresentative. Policy support may be a possible reason for the increased availability, indicating the important and positive role of pharmaceutical policies in improving the availability of EMC. A survey of 37 European countries from 2016 to 2018 showed that children and adolescents with cancer still experienced lack of access to essential medicines. [42] To address this issue, the Expert Committee in developing the WHO EMLc included several additional supportive care agents for cancer and the Essential Medicines Working Group of the International Society of Pediatric Oncology (SIOP) proposed a list of anti-neoplastic drugs, [43]–[44]. In our study, carboplatin, methotrexate, ifosfamide, cyclophosphamide, etoposide, vincristine, doxorubicin, dactinomycin, cytarabine, cisplatin are the top ten anticancer drugs in the research frequency and belong to the SIOP core catalog.

There were several limitations of this study. Firstly, limited by the number of studies included, economic regional and national availability rates estimated may not be stable or accurate. Secondly, limited by the quality of some studies, such as the medicines selected for investigation inconsistent, there were some bias in the results. However, this study provides a comprehensive map of the global, regional and national availability of EMC and that by medical institutions, original and generic medicines and medicine categories from 2009 to 2015 using evidence-based methodology, and there is nothing better for the moment, which shed light on the needs in practice and in research in the future.

## Conclusions

In conclusion, the availability rate of EMC is still low globally, with only marginal increase in the last decade and only a few categories reached reasonable and high availability (> 50%). Income is not always proportional to EMC. No country had reached the target of high availability rate (> 80%) of EMC. It is suggested to carry out relevant studies to fill the data gaps in the children’s essential medicine survey and improve the accessibility of children’s medicines. Substantial efforts from all stakeholders are warranted to improve the availability of EMC globally and its equity among countries. Continuous monitoring and timely reporting of the availability of EMC are also needed to facilitate targets setting and inform relevant policy making.

## Electronic supplementary material

Below is the link to the electronic supplementary material.

Supplementary Table 1. Search strategy. Supplementary Table 2. Quality assessment of included studiesusing the Joanna Briggs Institute?JBI?tool. Supplementary Table 3. The lists of essential medicines for children in included studies Supplementary Table 4. The global availability of essential medicines for children from 2009-2015. Supplementary Table 6. Characteristics of excluded studies

## Data Availability

All datasets generated and analyzed, including the study protocol, search strategy, list of the included and excluded studies, data extracted, quality assessment, merged data and investigated medicines of each study are available in the article and upon request from the corresponding author.

## List of abbreviations

ATC: Anatomical Therapeutic Chemical
CBM: China Biology Medicine
CI: Confidence Intervals
CNKI: China National Knowledge Infrastructure
EMLc: Essential Medicines for Children
HAI: Health Action International
HIC: High-income countries
JBI: Joanna Briggs Institute
LIC: Low income countries
LMC: Lower Middle income countries
MDGs: The United Nations Millennium Development Goals
NEML: National Essential Medicine List
NRHM: National Rural Health Mission’s list
PRISMA: The Preferred Reporting Items for Systematic Reviews and Meta-Analyses
SARA: Service availability and readiness assessment
SIOP: International Society of Pediatric Oncology
UMC: Upper-middle-income countries
WHA: World Health Assembly
WHO: World Health Organization
