# Supplementary material for: Global, regional and national availability of essential medicines for children, 2009–2020: a systematic review and meta-analysis
Source: BMC Public Health. 2023 Jun 20;23:1185. doi: 10.1186/s12889-023-15820-7 (PMC10280921; doi:10.1186/s12889-023-15820-7)
Supplement: Supplementary file 1 — Supplementary Table 1. Search strategy. Supplementary Table 2. Quality assessment of included studiesusing the Joanna Briggs Institute?JBI?tool. Supplementary Table 3. The lists of essential medicines for children in included studies Supplementary Table 4. The global availability of essential medicines for children from 2009-2015. Supplementary Table 6. Characteristics of excluded studies [file 12889_2023_15820_MOESM1_ESM.docx]

***Supplementary Material***

**Supplementary T****able 1. Search strategy**

| Search strategy for **PubMed** |
| --- |
| #1 "drugs, essential*"[MeSH Terms]  #2 "essential medicine*"[Title/Abstract] OR "essential drug*"[Title/Abstract] OR "essential medication*"[Title/Abstract]  #3 #1 OR #2 |
| Search strategy for **EMBASE (Ovid)** |
| #1 ("essential drug*" or "essential medicine*" or "essential medication*").kw,ti.  #2 exp essential drug/  #3 #1 or #2 |
| Search strategy for **Cochrane Central Register of Controlled Trials (CENTRAL)** |
| #1 ("essential drug*" or "essential medicine*" or "essential medication*").ti,kw,ab.  #2 exp Drugs, Essential/  #3 #1 or #2 |
| Search strategy for **Cochrane Reviews** |
| #1 "essential drug*" OR "essential medicine*" OR "essential medication*"  #2 MeSH descriptor: [Drugs, Essential] explode all trees  #3 #1 or #2 |
| Search strategy for **CNKI (Chinese)** |
| 高级检索  1 篇名：基本药物 + 基本药品  2 关键词：基本药物 + 基本药品  3 1 OR 2 |
| Search strategy for **CBM (Chinese)** |
| 1 ["基本药物"[中文标题:智能]](http://182.150.59.104:8888/http/77726476706e69737468656265737421e7e056d23439665f730d8de299566d36ad/zh/javascript:this.top.vpn_inject_scripts_window(this);vpn_eval((function%20()%20%7b%20toDoRelimitSearch();%20%7d).toString().slice(14,%20-2))) |
| Search strategy for **World Health Organization (Website)** |
| #1 "essential drug*" OR "essential medic*” |
| Search strategy for **Health Action International (Website)** |
| https://haiweb.org/what-we-do/price-availability-affordability/price-availability-data/ |

**Supplementary Table 2.** **Quality assessment of included studiesusing the [Joanna Briggs Institute](https://joannabriggs.org/)（JBI）tool**

| **First Author, year** | 1. **Was the sample frame appropriate to address the target population?** | 1. **Were study participants sampled in an appropriate way?** | 1. **Was the sample size adequate?** | 1. **Were the study subjects and the setting described in detail?** | 1. **Was the data analysis conducted with sufficient coverage of the identified sample?** | 1. **Were valid methods used for the identification of the condition?** | 1. **Was the condition measured in a standard, reliable way for all participants?** | 1. **Was there** **appropriate statistical analysis?** | 1. **Was the response rate adequate, and if not, was the low response rate managed appropriately?** | **Overall scores** | **Risk of bias** |
| --- | --- | --- | --- | --- | --- | --- | --- | --- | --- | --- | --- |
| Balasubramaniam, 2011 | Y | Y | U | Y | Y | Y | Y | Y | Y | 9 | L |
| Gitanjali, 2011 | Y | U | U | Y | N | Y | U | Y | Y | 8 | L |
| Anson, 2012 | Y | Y | U | Y | U | Y | Y | Y | Y | 9 | L |
| Wang, 2014 | Y | Y | U | Y | U | Y | Y | Y | U | 9 | L |
| Swain, 2015 | Y | Y | U | Y | U | Y | Y | U | Y | 9 | L |
| Sado, 2015 | Y | Y | U | Y | U | Y | Y | U | Y | 9 | L |
| Dorj, 2018 | Y | Y | U | Y | N | Y | Y | U | Y | 8 | L |
| Sun, 2018 | Y | N | U | Y | U | Y | Y | Y | U | 8 | L |
| Li, 2018 | Y | U | U | Y | N | Y | Y | Y | U | 8 | L |
| Droti, 2019 | U | Y | U | U | N | Y | Y | Y | Y | 8 | L |
| Faruqui, 2019 | Y | Y | U | Y | U | Y | U | Y | Y | 9 | L |
| Orubu, 2019 | Y | Y | Y | Y | U | Y | U | Y | Y | 9 | L |
| Wei, 2019 | Y | U | U | Y | Y | Y | Y | Y | U | 9 | L |
| Martei, 2020 | Y | U | U | Y | U | U | N | Y | Y | 8 | L |
| Wang, 2020 | Y | N | U | Y | U | Y | Y | Y | U | 8 | L |
| Dinh, 2021 | Y | Y | U | Y | Y | Y | Y | Y | Y | 9 | L |
| Mensah, 2021 | Y | N | U | Y | Y | U | Y | Y | Y | 8 | L |
| Tadesse, 2021 | Y | Y | Y | Y | Y | Y | Y | Y | Y | 9 | L |
| Dai, 2020 | Y | N | U | Y | Y | N | N | Y | Y | 6 | M |
| Wang Xiao, 2014 | Y | N | Y | Y | Y | Y | Y | Y | Y | 8 | L |
| Balasubramaniam, 2014 | Y | U | U | Y | U | Y | U | U | Y | 9 | L |
| Pujari, 2016 | Y | Y | U | Y | U | N | N | Y | Y | 7 | L |

*Note: 1. (1) Consider the purpose of the study; (2) Describe the scope of the surveyed institutions, including public and private sectors, hospitals, pharmacies and primary health centers; (3) Judged by the purpose of each study. If the above conditions are satisfied, the result is Y; if it is missing, it is N; if it is not described, it is U.*

*2. (1) The sampling method should be specified, if it is randomly selected, it should be Y; (2) If the sampling method is specified, but it is not random, it should be N; (3) If the sampling method is not described, it should be U*

*3. (1) If all institutions in the target area are investigated, it is Y; (2) If it is a medical institution selected with a reasonable explanation, it is Y; (3) If following standardised WHO/HAI methodology, It is Y; (4) If there is an alternative pharmacy, it is Y; (5) If the sample size is pre-estimated and does not meet the requirements, it is N; (6) If none of the above conditions are met, it is U.*

*4. If the type and the corresponding number of medical institutions are clearly described, it is Y; if lacking one of them, it is N; if it is not described in the text, it is U.*

*5.* *Whether the response rate of each group is balanced and whether the subgroup and overall response rates are different. If there are only subgroups, the differences between different subgroups are compared, and if the difference is within 30%, it is Y; if the difference is more than 30%, it is N; if not described, it is U.*

*6. (1) The report uses the WHO/HAI (or adapted) method or designs what was used and verified, then Y; (2) If the adaption is unclear, it is N; (3) if there is no related description, it is U.*

*7. (1) If it is extracted from the database or surveyed the same institutions by two investigators on the same day, it is Y; (2) if the survey is performed with questionnaire interview and staff cross check after training, it is Y; (3) if only one of above is satisfied, the result is Y; (4) if there are inconsistent and unreliable measurements, it is N; (5) if there is no description of the authenticity and consistency of data processing, it is U.*

*8. (1) If the definition of the availability rate is clearly described, it is Y; (2)* *if there is no description, it is U.*

*9. (1) There is a sufficiently high response rate that any difference is unlikely to affect the outcome (>=75%), Y; (2) if the response rare <75%, it is N; (3) if there is no description, it is U.*

**Supplementary Table 3. The lists of essential medicines for children in included studies**

| **Study ID** | **Medicine name** | **Strength** | **Dosage form** |
| --- | --- | --- | --- |
| Balasubramaniam, 2011 | Amoxicillin | 125 mg/5ml(100 ml) | Suspension |
|  | Amoxicillin + clavulanic acid | 125 mg + 31.25 mg/5 ml(100 ml) | Suspension |
|  | Beclometasone – MDI | 50 microgram/dose (200 doses) | Inhaler |
|  | Carbamazepine | 100 mg/5 ml (100 ml) | Suspension |
|  | Ceftriaxone | 1 gram (Vial) | Injection |
|  | Chlorphenamine | 2 mg/5 ml (100 ml) | Syrup |
|  | Clotrimazole | 1% (15 g tube) | Topical cream |
|  | Cloxacillin | 125 mg/5 ml (100 ml) | Syrup |
|  | Co-trimoxazole | 200 mg + 40 mg/5 ml (100 ml) | Suspension |
|  | Diazepam | 5 mg/ml (2 ml ampoule) | Injection |
|  | Diethylcarbamazine citrate | 50 mg | Tablet |
|  | Domperidone | 5 mg/5ml (100 ml) | Syrup |
|  | Erythromycin | 125 mg/5 ml (100 ml) | Syrup |
|  | Ferrous salt | 30 mg/ml (250 ml) | Suspension |
|  | Ibuprofen | 100 mg/5ml (100 ml) | Syrup |
|  | Mebendazole | 100 mg (6 tablets) | Chewable tablet |
|  | Mebendazole | 100 mg/5 ml (30 ml) | Syrup |
|  | Metronidazole | 200 mg | Tablet |
|  | Oral rehydration salt | Packet to make 1 litre of solution | Powder |
|  | Paracetamol | 120 mg/5 ml (100 ml) | Syrup |
|  | Paracetamol | 500 mg | Tablet |
|  | Salbutamol – MDI | 100 microgram/dose (200 doses) | Inhaler |
|  | Salbutamol | 0.5% (10 ml) | Respiratory solution |
|  | Vitamin C | 100 mg | Tablet |
| Gitanjali, 2011 | Cotrimoxazole | / | Syrup |
|  | Oral rehydration salt | / | Powder |
|  | Paracetamol | / | Syrup |
|  | Vitamin A | / | Solution |
|  | Zinc sulphate | / | Oral liquid/Tablet |
| Anson, 2012 | Salbutamol | 100 mcg/dose | Inhaler |
|  | Beclomethasone | 100 mcg/dose | Inhaler |
|  | Zinc | 20 mg | Dispersible tablet |
|  | Oral rehydration salt | 500 ml | Solution |
|  | Oral rehydration salt | To make 500ml | Powder |
|  | Amoxicillin | 100 mg/ml | Pediatric drops |
|  | Amoxicillin | 125 mg/5ml | Suspension |
|  | Amoxicillin | 250 mg/5ml | Suspension |
|  | Amoxicillin + Clavulanic Acid | 25 mg + 31.25mg/5ml | Suspension |
|  | Amoxicillin + Clavulanic Acid | 250 mg + 62.5mg/5ml | Suspension |
|  | Benzathine Penicillin G | 1.2 M units/Vial | Injection |
|  | Ceftriaxone | 500 mg/Vial | Injection |
|  | Chloramphenicol | 1 g/Vial | Injection |
|  | Cotrimoxazole (Trimethoprin + Sulfamethoxazole) | 8 + 40 mg/ml | Suspension |
|  | Gentamicin | 10mg/ml | Injection |
|  | Procaine Penicillin G | 4 M units/Vial | Injection |
|  | Primaquine | 15mg | Capsule/Tablet |
|  | Ibuprofen | 200 mg | Capsule/Tablet |
|  | Paracetamol | 25 mg/ml | Syrup/Suspension |
|  | Morphine Sulfate | 10mg/ml | Injection |
|  | Vitamin A | 50,000 units | Capsule/Tablet |
|  | Isoniazid | 100 mg | Capsule/Tablet |
|  | Ferrous Salt | 125 mg/5ml | Suspension |
|  | Diazepam | 5 mg/ml | Injection |
|  | Carbamazepine | 100 mg/5ml | Suspension |
|  | Phenobarbital | 3mg/ml | Oral liquid |
|  | Phenytoin | 25 or 30mg/5ml | Suspension |
| Wang, 2014 | Amoxicillin | 250 mg | Capsule/Tablet |
|  | Amoxicillin/ Clavulanic acid | 125 mg+31.25 mg/5 ml | Suspension |
|  | Azithromycin | 250 mg | Tablet |
|  | Benzylpenicillin | 1 million IU | Injection |
|  | Calamine | 100 ml | Lotion |
|  | Carbamazepine | 200 mg | Tablet |
|  | Cefazolin | 1 g | Injection |
|  | Ceftriaxone | 250 mg | Injection |
|  | Chloramphenicol | 250 mg | Tablet |
|  | Chlorpheniramine | 4 mg | Tablet |
|  | Diazepam | 5 mg/ml | Injection |
|  | Fluconazole | 50 mg | Capsule |
|  | Ibuprofen | 200 mg | Tablet |
|  | Isoniazid | 100 mg | Tablet |
|  | Morphine | 10 mg | Tablet |
|  | Oral rehydration salt | 500 ml | Oral solution |
|  | Paracetamol | 500 mg | Tablet |
|  | Phenobarbital | 30 mg | Tablet |
|  | Phenytoin | 50 mg | Tablet |
|  | Procaine penicillin | 600 mg | Injection |
|  | Salbutamol | 100 mcg/dose | Inhaler |
|  | Albendazole | 200 mg | Tablet |
|  | Aminophylline | 25 mg/ml | Injection |
|  | Amoxicillin/ Clavulanic acid | 250 mg+125 mg | Tablet |
|  | Beclomethasone | 50 mcg/ dose | Inhaler |
|  | Phenobarbital | 100 mg/ml | Injection |
|  | Vitamin A | 25,000 IU | Capsule |
|  | Vitamin B6 | 50 mg/ml | Injection |
| Swain, 2015 | Albendazole | 200 mg/5 ml | Suspension |
|  | Amoxicillin | 125 mg/ml | Suspension |
|  | Amoxicillin | 250 mg | Dispersible scored tablet |
|  | Amoxicillin + clavulanic acid | 125mg + 31.25mg | Dry syrup |
|  | Amoxicillin + clavulanic acid | 250 mg + 125 mg | Dispersible kid forte, FC tablet |
|  | Artemether + lumefantrine | 20mg + 120mg | Dispersibletablet |
|  | Beclomethasone | 100 µg/dose | Inhaler |
|  | Benzyl benzoate | 25% | Lotion |
|  | Benzylpenicillin | 600 mg = 1 million IU | Injection |
|  | Carbamazepine | 100 mg/5 ml | Suspension |
|  | Carbamazepine | 100 mg | Chewable tablet |
|  | Chloramphenicol | 500 mg/Vial | Injection |
|  | Chloroquine | 50 mg/5 ml | Suspension |
|  | Co-trimoxazole | 100 mg + 20 mg (also expressed as 400 mg + 80 mg) | Dispersible tablet |
|  | Diazepam | 5 mg/ml | Rectal solution |
|  | Ferrous sulfate | 50 mg Fe/5 ml | Suspension |
|  | Gentamycin | 10 mg/ml | Injection |
|  | Ibuprofen | 200 mg | Tablet |
|  | Isoniazid + rifampicin + pyrazinamide | 50 mg + 100 mg + 300 mg | Dispersible tablet |
|  | Oral rehydration salt | 200 ml | Solution Sachets |
|  | Oral rehydration salt | 1L | Solution Sachets |
|  | Paracetamol | 120 mg/5 ml or 125 mg/5 ml | Suspension |
|  | Paracetamol | 250 mg | Scored tablet |
|  | Phenobarbital | 200 mg/ml | Injection |
|  | Phenytoin | 25 or 30 mg/ml | Suspension |
|  | Procaine penicillin | 1 g = 1 million IU | Injection |
|  | Salbutamol | 100 mcg/dose | Inhaler |
|  | Vitamin A | 25 000 IU | Capsule |
|  | Zinc | 20 mg | Dispersible tablet |
|  | Prednisolone | 5 mg/5 ml | Suspension |
|  | Azithromycin | 250 mg | Dispersible tablet |
|  | Ofloxacin | 200 mg | Tablet |
|  | Ondansetron | 2 mg/5 ml | Syrup/suspension |
|  | Valproic acid | 200 mg/5 ml | Oral liquid |
| Sado, 2015 | Amoxicillin | 125 mg | Suspension |
|  | Amoxicillin | 250 mg | Suspension |
|  | Amoxicillin + clavulanic acid | 125 mg | Suspension |
|  | Amoxicillin + clavulanic acid | 250 mg | Suspension |
|  | Artesunate | 60 mg | Vial |
|  | Artemether + Lumefantrine | 20 mg + 120 mg | Dispersible tablet |
|  | Chloramphenicol | 1gm | Vial |
|  | Carbamazepine | 100 mg/5 ml | Syrup |
|  | Ceftriaxone | 500 mg | Vial |
|  | Cotrimoxazole | 40 mg + 200 mg/5 ml | Suspension |
|  | Diazepam | 5 mg/ml | Ampoule |
|  | Gentamicin | 20 mg/2 ml | Ampoule |
|  | Ibuprofen | 100 mg/5 ml | Suspension |
|  | Oral rehydration salt | To make 500ml | Solution |
|  | Oral rehydration salt | To make 1000ml | Solution |
|  | Paracetamol | 120 mg/5 ml | Syrup |
|  | Paracetamol | 125 mg | Suppository |
|  | Penicillin G | 1 million IU | Vial |
|  | Procaine Penicillin G | 4 million IU | Vial |
|  | Salbutamol | 100 mcg/dose | Inhaler |
|  | Vitamin A | 50,000 units | Capsule |
|  | Zinc Phosphate | / | / |
| Dorj, 2018 | Albendazole | 200 mg | Tablet |
|  | Amoxicillin | 250 mg | Capsule |
|  | Amoxicillin | 125 mg | Suspension |
|  | Aminophylline | 25 mg/ml | Injection |
|  | Amoxicillin + clavulanic acid | 125 mg + 125 mg | Tablet |
|  | Amoxicillin + clavulanic acid | 125 mg + 31.25 mg | Suspension |
|  | Amoxicillin + clavulanic acid | 250 mg + 31.25 mg | Suspension |
|  | Azithromycin | 250 mg | Capsule |
|  | Azithromycin | 200 mg/ 5ml | Suspension |
|  | Beclomethasone | 50 mg/day | Inhaler |
|  | Benzylpenicillin | 600 mg = 1 million IU | Injection |
|  | Carbamazepine | 100 mg | Tablet |
|  | Carbamazepine | 100 mg/5 ml | Suspension |
|  | Cefazolin | 1 g, vial | Injection |
|  | Ceftriaxone | 500 mg, vial | Injection |
|  | Chloramphenicol | 250 mg | Tablet |
|  | Chloramphenicol | 1 g, vial | Injection |
|  | Chlorpheniramine | 4 mg | Tablet |
|  | Clarithromycin | 125 mg/ 5 ml | Suspension |
|  | Clarithromycin | 125 mg | Tablet |
|  | Cotrimoxazole | 100 mg + 20 mg (also expressedas 400 mg + 80 mg) | Tablet |
|  | Cotrimoxazole | 100 ml | Suspension |
|  | Diazepam | 5 mg | Tablet |
|  | Ferrous salt | 30 mg/5 ml | Suspension |
|  | Fluconazole | 150 mg | Capsule |
|  | Gentamycin | 10 mg/ml | Injection |
|  | Ibuprofen | 2 mg | Tablet |
|  | Ibuprofen | 100 ml | Suspension |
|  | Isoniazide | 100 mg | Tablet |
|  | Morphine | 10 mg | Tablet |
|  | Morphine | 10 mg/5 ml | Oral solution |
|  | Oral rehydration salt | 500 ml | Solution Sachets |
|  | Paracetamol | 250 mg | Tablet |
|  | Paracetamol | 125 mg | Suppository |
|  | Paracetamol | 250 mg | Suppository |
|  | Paracetamol | 125 mg | Suspension |
|  | Phenobarbital | 30 mg | Tablet |
|  | Phenobarbital | 100 mg/ml | Injection |
|  | Phenytoin | 50 mg | Tablet |
|  | Phenytoin | 25 mg, 30 mg/5 ml | Suspension |
|  | Procain penicillin | 1 g, vial | Injection |
|  | Salbutamol | 100 mcg | Inhaler |
|  | Vitamin A | 100.000 IU | Capsule |
|  | Vitamin B6 | 50 mg/ml | Injection |
|  | Zinc | 20 mg | Tablet |
| Sun, 2018 | Aciclovir | 200 mg | Capsule/Tablet |
|  | Amoxicillin | 250 mg | Capsule/Tablet |
|  | Amoxicillin + clavulanic acid | 125/31.25 mg/5 ml | Suspension |
|  | Azithromycin | 250 mg | Capsule/Tablet |
|  | Calamine | 100 mL | Lotion |
|  | Calcium gluconate | 100 mg/mL | Injection |
|  | Carbamazepine | 200 mg | Tablet |
|  | Ceftriaxone | 1 g/phial | Injection |
|  | Ceftazidime | 1 g/phial | Injection |
|  | Clarithromycin (sustained-release) | 500 mg | Tablet |
|  | Clindamycin | 150 mg | Capsule |
|  | Diazepam | 5 mg/mL | Injection |
|  | Fluconazole | 50 mg | Capsule/Tablet |
|  | Folic Acid | 5 mg | Tablet |
|  | Furosemide | 10 mg/mL | Injection |
|  | Hydrochlorothiazide | 25 mg | Tablet |
|  | Hydrocortisone | 100 mg/phial | Injection |
|  | Ibuprofen | 200 mg | Tablet |
|  | Loratadine | 10 mg | Tablet |
|  | Miconazole nitrate | 2% | Cream |
|  | Mupirocin | 2% | Cream |
|  | Omeprazole (enteric-coated) | 20 mg | Capsule/Tablet |
|  | Paracetamol | 500 mg | Tablet |
|  | Phenobarbital | 30 mg | Tablet |
|  | Phenytoin | 100 mg | Tablet |
|  | Propylthiouracil | 50 mg | Tablet |
|  | Ranitidine | 150 mg | Tablet |
|  | Salbutamol | 100 μg/dose | Inhaler |
|  | Sodium valproate | 200 mg | Tablet |
|  | Aminophylline | 100 mg | Tablet |
|  | Amoxicillin + clavulanic acid | 1000/200 mg/phail | Injection |
|  | Cefuroxime | 250 mg | Tablet |
|  | Chlorphenamine maleate | 4 mg | Tablet |
|  | Dexamethasone | 5 mg/mL | Injection |
|  | Clarithromycin | 250 mg | Tablet |
|  | Ibuprofen | 100 mg/5 mL | Suspension |
|  | Phenobarbital | 100 mg/mL | Injection |
|  | Vitamin B6 | 50 mg/mL | Injection |
|  | Vitamin C | 100 mg | Tablet |
|  | Sodium valproate (sustained-release) | 500 mg | Tablet |
| Li, 2018 | Salbutamol | / | / |
|  | Ceftriaxone | / | / |
|  | Ibuprofen | / | / |
|  | Metformin | / | / |
|  | Amoxicillin | / | / |
|  | Sodium Valproate | / | / |
|  | Albendazole | / | / |
|  | Metronidazole | / | / |
|  | Enalapril | / | / |
|  | Hydrochlorothiazide | / | / |
|  | Cephalexin | / | / |
|  | Diazepam | / | / |
|  | Ranitidine | / | / |
|  | Amitriptyline | / | / |
| Droti, 2019 | Amoxicillin | / | Syrup/Suspension |
|  | Amoxicillin | / | Powder for injection |
|  | Ceftriaxone | / | Powder for injection |
|  | Gentamincin | / | Injection |
|  | Procaine benzylpenicillin | / | Injection |
|  | Oral rehydration salt | / | Solution Sachets |
|  | Zinc | / | Tablet |
|  | Artemisinin-based combination therapy | / | / |
|  | Artesunate | / | Rectal or injectable forms |
|  | Vitamin A | / | Capsule |
|  | Morphine | / | Granule, injection, capsules or tablets |
|  | Paracetamol | / | Syrup/Suspension |
| Faruqui, 2019 | Asparaginase | 10000 IU in vial | Powder injection |
|  | Bleomycin | 15 mg | Powder injection |
|  | Carboplatin | 150 mg/15 ml | Injection |
|  | Carboplatin | 450 mg/45 ml | Injection |
|  | Carboplatin | 50 mg/5 mL | Injection |
|  | Carboplatin | 600 mg/60 ml | Injection |
|  | Carboplatin | no specific strength | / |
|  | Cisplatin | 100 mg/100 m1 | Injection |
|  | Cisplatin | 50 mg/50 mL | Injection |
|  | Cisplatin | no specific strength | / |
|  | Cytarabine | 100 mg in vial | Powder injection |
|  | Dacarbazine | 100 mg in vial | Powder injection |
|  | Dactinomycin | 500 μg in vial | Powder injection |
|  | Daunorubicin | 50 mg in vial | Powder injection |
|  | Dexamethasone | 2 mg/5 ml | Oral liquid |
|  | Doxorubicin | 10 mg | Powder injection |
|  | Doxorubicin | 50 mg | Powder injection |
|  | Doxorubicin | no specific strength | / |
|  | Etoposide | 100 mg | Capsule |
|  | Etoposide | 100 mg | Injection |
|  | Ifosfamide | 500 mg | Powder injection |
|  | Ifosfamide | 2 g vial | Powder injection |
|  | Ifosfamide | 1 g | Powder injection |
|  | Ifosfamide | no specific strength | Powder injection |
|  | Mercaptopurine | 50 mg | Tablet |
|  | Methotrexate | 50 mg | Powder injection |
|  | Methotrexate | 2.5 mg | Tablet |
|  | Paclitaxel | 6 mg/mL | Powder injection |
|  | Prednisolone | 5 mg/mL | Oral liquid |
|  | Prednisolone | 25 mg | Tablet |
|  | Prednisolone | 5 mg | Tablet |
|  | Prednisolone | no specific strength | Tablet |
|  | Thioguanine | 40 mg | Solid oral dosage form |
|  | Vinblastine | 10 mg (sulfate) in vial | Powder injection |
|  | Vincristine | 1 mg | Powder injection |
|  | Vincristine | 5 mg | Powder injection |
|  | Vincristine | no specific strength | Powder injection |
|  | Amoxicillin | 250 mg | Capsule/Tablet |
|  | Ciprofloxacin | 500 mg | Capsule/Tablet |
|  | Metformin | 500 mg | Capsule/Tablet |
|  | Omeprazole | 20 mg | Capsule/Tablet |
| Orubu, 2019 | Digoxin | / | Tablet |
|  | Digoxin | / | Injection |
|  | Digoxin | / | Oral liquid |
|  | Dopamine | / | Injection |
|  | Enalapril | / | Tablet |
|  | Furosemide | / | Tablet |
|  | Furosemide | / | Injection |
|  | Furosemide | / | Oral liquid |
|  | HCT | / | Tablet |
|  | Mannitol | / | / |
|  | Spironolactone | / | Tablet |
|  | Spironolactone | / | Oral liquid |
| Wei, 2019 | Amitriptyline | 25 mg | / |
|  | Azithromycin | 0.25 g | / |
|  | Atenolol | 50 mg | / |
|  | Aminophylline | 0.1 g | / |
|  | Amlodipine | 5 mg | / |
|  | Omeprazole | 20 mg | / |
|  | Omeprazole | 40 mg | / |
|  | Phenytoin sodium | 0.1 g | / |
|  | Beclometasone Dipropionate | 50 μg/vial, 200 vial | / |
|  | Sodium Valproate | 0.2 g | / |
|  | Ibuprofen | 0.2 g | / |
|  | Metformin | 0.5 g | / |
|  | Erythromycin | 0.25 g | / |
|  | Ciprofloxacin | 0.25 g | / |
|  | Metronidazole | 0.2 g | / |
|  | Clarithromycin | 0.25 g | / |
|  | Chlorphenamine | 4 mg | / |
|  | Loratadine | 10 mg | / |
|  | Hydrocortisone | 0.1 g | / |
|  | Cefalexin | 0.25 g | / |
|  | Cefuroxime | 0.25 g | / |
|  | Ceftriaxone | 0.25 g | / |
|  | Nifedipine | 10 mg | / |
|  | Simvastatin | 20 mg | / |
|  | Enalapril | 5 mg | / |
|  | Promethazine | 25 mg | / |
|  | Albendazole | 0.2 g | / |
|  | Amoxicillin | 0.25 g | / |
|  | Amoxicillin and Clavulanate Potassium | 0.375 g | / |
|  | Amoxicillin and Clavulanate Potassium | 1.0 g | / |
|  | Aspirin | 0.3 g | / |
|  | Paracetamol | 0.5 g | / |
|  | Captopril | 25 mg | / |
|  | Ranitidine | 0.15 g | / |
|  | Salbutamol | 100 μg/vial, 200 vial | / |
|  | Cefuroxim | 0.75 g | / |
|  | Ceftriaxone | 1.0 g | / |
|  | Vitamin K1 | 1 ml：10 mg | / |
|  | Isosorbide Dinitrate | 5 mg | / |
|  | Enalapril | 10 mg | / |
|  | Promethazine | 1 ml：25 mg | / |
|  | Isoniazid | 0.1 g | / |
|  | Carbamazepine | 0.2 g | / |
|  | Diclofenac sodium | 25 mg | / |
|  | Cefuroxime | 0.75 g | / |
| Martei, 2020 | Asparaginase | / | Injection |
|  | Bleomycin | / | Injection |
|  | Carboplatin | / | Injection |
|  | Cisplatin | / | Injection |
|  | Cyclophosphamide | / | Tablet |
|  | Cyclophosphamide | / | Injection |
|  | Cytarabine | / | Injection |
|  | Dacarbazine | / | Injection |
|  | Dactinomycin | / | Injection |
|  | Daunorubicin | / | Injection |
|  | Doxorubicin | / | Injection |
|  | Etoposide | / | Capsule |
|  | Etoposide | / | Injection |
|  | Hydroxycarbamide | / | Tablet/Capsule |
|  | Ifosfamide | / | Injection |
|  | Mercaptopurine | / | Tablet |
|  | Methotrexate | / | Tablet |
|  | Methotrexate | / | Injection |
|  | Thioguanine | / | Tablet |
|  | Vinblastine | / | Injection |
|  | Vincristine | / | Injection |
|  | 13-cis retinoic acid | / | Tablet/Capsule |
|  | All-trans retinoic acid | / | Capsule |
|  | Busulphan | / | Tablet |
|  | Imatinib | / | Tablet |
|  | Irinotecan | / | Injection |
|  | Melphalan | / | Tablet |
|  | Topotecan | / | Injection |
|  | Vinorelbine | / | Injection |
|  | Calcium folinate (leucovorin) | / | Injection |
|  | Calcium folinate (leucovorin) | / | Tablet |
|  | Filgrastim | / | Injection |
|  | Mesna | / | Tablet |
|  | Mesna | / | Injection |
| Wang, 2020 | Epinephrine | 1 mL∶1 mg | Injection |
|  | Amoxicillin | 250 mg | Tablet/Capsule |
|  | Azithromycin | 250 mg | Tablet/Capsule |
|  | Calamite | 100 ml | Lotion |
|  | Carbamazepine | 200 mg | Tablet |
|  | Cefazolin | 1 g/stick | Injection |
|  | Ceftriaxone | 1 g/stick | Injection |
|  | Diazepam | 2 mL∶10 mg | Injection |
|  | Fluconazole | 100 mL∶0.2 g | Injection |
|  | Furosemide | 2 mL∶20 mg | Injection |
|  | Hydrochlorothiazide | 25 mg | Tablet |
|  | Ibuprofen | 200 mg | Tablet/Capsule |
|  | Isoniazid | 100 mg | Tablet |
|  | Levothyroxine | 50 mcg | Tablet |
|  | Loratadine | 10 mg | Tablet/Capsule |
|  | Mannitol | 250 mL∶50 g | Injection |
|  | Metformin | 500 mg | Tablet/Capsule |
|  | Metronidazole | 200 mg | Tablet/Capsule |
|  | Midazolam | 1 mL∶5 mg | Injection |
|  | Paracetamol | 500 mg | Tablet |
|  | Phenobarbital | 30 mg | Tablet |
|  | Phenytoin | 50 mg | Tablet |
|  | Piperacillin tazobactam | 4.5 g/stick | Injection |
|  | Salbutamol | 100 mcg/vial | Aerosol |
|  | Albendazole | 200 mg | Tablet |
|  | Aspirin | 100 mg | Tablet |
|  | Cefuroxime | 750 mg | Injection |
|  | Digoxin | 2 mL∶0.5 mg | Injection |
|  | Prednisone | 5 mg | Tablet |
|  | Vitamin K1 | 1 mL∶10 mg | Injection |
| Dinh, 2021 | Amoxicillin | 125 mg/5 mL | / |
|  | Amoxicillin + clavulanic acid | 125+31.25 mg/5 mL | / |
|  | Beclometasone | 100 μg/dose | / |
|  | Benzylpenicillin | 1 MIU (600 mg) | Vial |
|  | Ceftriaxone | 500 mg | Vial |
|  | Chloramphenicol | 1 g | Vial |
|  | Ferrrous salt | 30 mg Fe/5 mL | / |
|  | Ibuprofen | 200 mg | Tablet/Capsule |
|  | Oral rehydration salt | to make 1 L | Sachet |
|  | Paracetamol | 24 mg/mL | Tablet/Capsule |
|  | Procaine benzylpenicillin | 1 g (1 MIU) | Vial |
|  | Salbutamol | 100 μg/dose | / |
|  | Vitamin A | 100 000 IU | Tablet/Capsule |
|  | Zinc | 20 mg | Tablet/Capsule |
|  | Albendazole | 400 mg | Tablet/Capsule |
|  | Amoxicillin + clavulanic acid | 250+31.25 mg* | Sachet |
|  | Carbamazepine | 200 mg | Tablet/Capsule |
|  | Co-trimoxazole | 80+400 mg | Sachet |
|  | Co-trimoxazole | 8+40 mg/mL | Tablet/Capsule |
|  | Diazepam | 5 mg | Tablet/Capsule |
|  | Gentamicin | 40 mg/mL | / |
|  | Ibuprofen | 100 mg/5 mL | / |
|  | Mebendazole | 500 mg | Tablet/Capsule |
|  | Morphine | 10 mg/mL | / |
|  | Oral rehydration salt | to make 200 mL | Sachet |
|  | Paracetamol | 150 mg | Sachet |
|  | Paracetamol | 500 mg | Tablet/Capsule |
|  | Phenobarbital | 100 mg/mL | / |
| Mensah, 2021 | Dactinomycin | 500 μg in vial | Powder injection |
|  | L-Asparaginase | 10,000 IU in via | Powder injection |
|  | Carboplatin | 150 mg/15 mL | Injection |
|  | Carboplatin | 450 mg/45 mL | Injection |
|  | Etoposide | 100 mg/5 mL | Injection |
|  | Ifosphamide | 1 g in vial | Powder injection |
|  | Bleomycin | 30 IU | Injection |
|  | Cyclophosphamide | 50 mg in vial | Powder injection |
|  | Vincristine | 1 mg in vial | Powder injection |
|  | 6-Mecarptopurine | 50 mg | Tablet |
|  | Dacarbazine | 200 mg in vial | Powder injection |
|  | 5-Florouracil | 500 mg/10 ml | Injection |
|  | Methotrexate | 50 mg in ViaL | Injection |
|  | Methotrexate | 2.5 mg | Tablet |
|  | Leucovorin | 30 mg in vial | Injection |
|  | Procarbazine | 50 mg | Tablet |
|  | Chlormbucil | 2 mg | Tablet |
|  | Cytarabine | 100 mg in vial | Injection |
|  | Granisetron | 1 mg in vial | Injection |
|  | Dexamethasone | 0.5 mg | Tablet |
|  | Dexamethasone | 4 mg | Tablet |
|  | Dexamethasone | 8 mg/2 ml | Injection |
|  | Prednisolone | 5 mg | Tablet |
|  | Hydrocortisone | 100 mg in vial | Injection |
|  | Allopurinol | 100 mg | Tablet |
|  | Allopurinol | 300 mg | Tablet |
|  | Nexium | 10 mg | Powder |
| Tadesse, 2021 | Amoxicillin | 125 mg/ml | Suspension |
|  | Amoxicillin | 250 mg | Dispersible tablet |
|  | Amoxicillin+Clavulanic acid | 125 + 31.25 mg/5 ml | Suspension |
|  | Amoxicillin+Clavulanic acid | 125 mg + 31.25 mg | Dispersible tablet |
|  | Ampicillin | 500 mg | Injection |
|  | Artemether +Lumefantrine | 20 mg + 120 mg | Tablet |
|  | Artesunate | 60 mg | Injection |
|  | Beclomethasone inhaler | 100mcg/dose | Inhaler |
|  | Benzylpenicillin | 1MIU | Powder |
|  | Carbamazepine | 100 mg/5 ml | Suspension |
|  | Ceftriaxone injection | 1 g | Powder |
|  | Chloramphenicol injection | 1 g | Powder |
|  | Cloxacillin | 125 mg/5 ml | Suspension |
|  | Cotrimoxazole (Sulphamethoxazole + Trimethoprim) | 200 mg + 40 mg/5 ml | Suspension |
|  | Diazepam | 5 mg/ml | Solution |
|  | Ferrous salt | 30 mg Fe/5 ml | Suspension |
|  | Gentamycin | 40 mg/ml | Injection |
|  | Ibuprofen | 100 mg/5 ml | Syrup |
|  | Isoniazide | 100 mg | Tablet |
|  | Morphine | 10 mg/5 ml | Oral Solution |
|  | Oral Rehydration Solution | 1 litter | Powder |
|  | Paracetamol | 120 mg/5 ml | Syrup |
|  | Paracetamol | 125 mg | Suppository |
|  | Penicillin G, Benzathine penicillin | 1.2MIU | Injection |
|  | Phenobarbitone | 30 mg | Syrup |
|  | Phenytoin | 50 mg | Suspension |
|  | Procaine penicillin injection | 1 MIU | Powder |
|  | Salbutamol Puff | 100mcg/dose | Inhaler |
|  | Vitamin A | 100,000 IU | Capsule |
|  | Zinc sulfate | 20 mg | Tablet |
| Dai, 2020 | Cefazoline | 1.0 g | Injection |
|  | Ceftriaxone | 0.5 g | Injection |
|  | Cefuroxime | 0.75 g | Injection |
|  | Cefuroxime | 0.25 g | Tablet |
|  | Cefotaxime | 0.5 g | Injection |
|  | Ceftadime | 1.0 g | Injection |
|  | Vancomycin | 0.5 g | Injection |
|  | Fluconazole | 2.0 mg/ml | Injection |
|  | Fluconazole | 50 mg | Tablet/Capsule |
|  | Acyclovir | 0.25 g | Injection |
|  | Acyclovir | 0.2 g | Tablet |
|  | Nystatin | 500000U | Tablet |
|  | Metronidazole | 0.5 g/100 ml | Injection |
|  | Metronidazole | 0.2 g | Tablet |
|  | Azithromycin | 0.25 g | Tablet |
|  | Isoniazid | 100 mg | Tablet |
|  | Rifampicin | 150 mg | Tablet |
|  | Carbamazepine | 0.2 g | Tablet |
|  | Phenobarbital | 30 mg | Tablet |
|  | Sodium valproate | 0.5 g | Tablet |
|  | Enalapril | 5 mg | Tablet |
|  | Digoxin | 50 μg/ml | Oral liquid |
|  | Digoxin | 0.25 mg | Tablet |
|  | Salbutamol | 100 µg/ gush | Aerosol |
|  | Montelukast | 5 mg | Montelukast |
|  | Cyclosporine | 25 mg | Tablet/Capsule |
|  | Cytarabine | 100 mg | Injection |
|  | Cytarabine | 500 mg | Injection |
|  | Ifosfamide | 1 g | Injection |
|  | Methotrexate | 2.5 mg | Tablet |
|  | Vincristine | 1 mg | Injection |
|  | Omeprazole | 10 mg | Tablet |
|  | Omeprazole | 10 mg | Tablet |
|  | Ranitidine | 150 mg | Tablet/Capsule |
|  | Aspirin | 0.1 g | Tablet |
|  | Mupirocin | 0.20% | Ointment |
|  | Miconazole | 0.20% | Ointment |
|  | Loratadine | 10 mg | Tablet |
|  | Loratadine | 1 mg/ml | Oral liquid |
|  | Prednisolone | 5 mg | Tablet |
|  | Ibuprofen | 200 mg/5ml | Oral liquid |
|  | Levothyroxine | 50 μg | Tablet |
| Wang Xiao, 2014 | Amoxicillin | 250 mg | Tablet/Capsule |
|  | Amoxicillin + clavulanic acid | 125 mg + 31.25 mg/5 ml | Suspension |
|  | Azithromycin | 250 mg | Tablet |
|  | Penicillin | 1 million IU | Injection |
|  | Calamine | 100 ml | Lotion |
|  | Carbamazepine | 200 mg | Tablet |
|  | Cefazolin | 1g/piece | Injection |
|  | Cefatriaxone | 250 mg/piece | Injection |
|  | Cold Granules for children | 12 g | Granule |
|  | Chlorphenamine maleate | 4 mg | Tablet |
|  | Fluconazol | 50 mg | Capsule |
|  | Isoniazid | 100 mg | Tablet |
|  | Oral Rehydration Salt | 500 ml | Oral liquid |
|  | Phenobarbital | 30 mg | Tablet |
|  | Procaine penicillin | 600 mg/piece | Injection |
|  | Albendazole | 200 mg | Tablet |
|  | Aminophylline | 25 mg/ml | Injection |
|  | Amoxicillin + clavulanic acid | 250 mg + 125 mg | Tablet |
|  | Beclometasone | 50 mcg/vial | Aerosol |
|  | Phenobarbital | 100 mg/ml | Injection |
|  | Vitamin A | 25000 IU | Capsule |
|  | Vitamin B | 50 mg/ml | Injection |
|  | Chloramphenicol | 250 mg | Tablet |
|  | Shuanghuanglian Oral Liquid | 10 ml | Oral Liquid |
|  | Diazepam | 5 mg/ml | Injection |
|  | Ibuprofen | 200 mg | Tablet |
|  | Morphine | 10 mg | Tablet |
|  | Paracetamol | 500 mg | Tablet |
|  | Phenytoin sodium | 50 mg | Tablet |
|  | Salbutamol | 100 mcg/ gush | Aerosol |
| Balasubramaniam, 2014 | Amoxicillin | 125 mg/5 mL (100 mL) | Suspension |
|  | Amoxicillin | 250 mg | Tablet/Capsule |
|  | Amoxicillin + clavulanic acid | 125 mg + 31.25 mg/5 mL (100 mL) | Suspension |
|  | Beclometasone -MDI | 100 microgram/dose (200 doses) | Inhaler |
|  | Carbamazepine | 100 mg/5 mL (100 mL) | Suspension |
|  | Ceftriaxone | 1 g (vial) | Injection |
|  | Chlorphenamine | 2 mg/5 mL (100 mL) | Syrup |
|  | Clotrimazole | 1 % (15 g tube) | Topical cream |
|  | Cloxacillin | 125 mg/5 mL (100 mL) | Syrup |
|  | Co-trimoxazole | 200 mg + 40 mg/5 mL (100 mL) | Suspension |
|  | Diazepam | 5 mg/mL (2 mL ampoule) | Injection |
|  | Diethylcarbamazine citrate | 50 mg | Tablet |
|  | Domperidone | 5 mg/5 mL (100 mL) | Syrup |
|  | Erythromycin | 125 mg/5 mL (100 mL) | Syrup |
|  | Ferrous salt | 30 mg/mL (250 mL) | Suspension |
|  | Ibuprofen | 100 mg/5 mL (100 mL) | Syrup |
|  | Mebendazole | 100 mg (6 tablets) | Chewable tablet |
|  | Mebendazole | 100 mg/5 mL (30 mL) | Syrup |
|  | Metronidazole | 200 mg | Tablet |
|  | Oral rehydration salt | Packet to make 1 L | Solution |
|  | Paracetamol | 120 mg/5 mL (100 mL) | Syrup |
|  | Paracetamol | 500 mg | Tablet |
|  | Salbutamol –MDI | 100 microgram/dose (200 doses) | Inhaler |
|  | Salbutamol | 0.5 % (10 mL) | Solution |
|  | Vitamin C | 100 mg | Tablet |
| Pujari, 2016 | Cotrimoxazole | / | Suspension |
|  | Amoxicillin + clavulanic acid | / | Suspension |
|  | Gentamicin | / | Injection |
|  | Azithromycin | / | Syrup |
|  | Ondansetron | / | Syrup/suspension |
|  | Oral rehydration solution | to make 1 L | / |
|  | Paracetamol | / | Suspension |
|  | Ibuprofen | / | Suspension |
|  | Vitamin A | / | / |
|  | Ferrous salt | / | Drops |

**Supplementary Table 4. The global availability of essential medicines for children from 2009-2015**

| **ATC** | **A** | **B** | **D** | **J** | **L** | **M** | **N** | **P** | **R** | **Overall** |
| --- | --- | --- | --- | --- | --- | --- | --- | --- | --- | --- |
| **Global** | **55.8** | **21.5** | **71.3** | **40.2** | **5.3** | **21.1** | **32.7** | **27.0** | **49.3** | **39.0** |
| **95%CI** | **47.4-64.0** | **3.6-46.6** | **31.6-98.3** | **34.9-45.5** | **1.4-11.0** | **5.8-41.4** | **23.7-42.3** | **17.8-37.1** | **35.3-63.4** | **35.5-42.5** |
| **World bank county-income groups** | | | | | | | | | | |
| **LIC** | **55.8** | **/** | **/** | **44.4** | **/** | **0.0** | **27.2** | **7.5** | **50.9** | **38.6** |
| **95%CI** | **43.7-67.6** | **/** | **/** | **36.2-52.7** | **/** | **0.0-3.1** | **14.2-42.6** | **4.2-11.6** | **37.4-64.4** | **32.8-44.7** |
| **LMC** | **61.1** | **21.5** | **71.8** | **46.3** | **5.3** | **33** | **56.1** | **45.9** | **68.7** | **48.1** |
| **95%CI** | **45.2-75.9** | **3.6-46.6** | **12.2-100.0** | **36.4-56.4** | **1.4-11.0** | **13.6-55.5** | **38.0-73.5** | **22.6-70.0** | **54.3-81.5** | **41.9-54.4** |
| **UMC** | **27.7** | **/** | **70.8** | **17.1** | **/** | **3.2** | **8.5** | **25.5** | **10.9** | **17.0** |
| **95%CI** | **5.8-57.4** | **/** | **62.3-78.7** | **8.9-27.2** | **/** | **0.5-7.4** | **2.5-17.1** | **3.3-58.2** | **4.2-19.9** | **12.1-22.5** |
| **Country** | | | | | | | | | | |
| **Sri Lanka** | **80.4** | **40.4** | **71.8** | **75.8** | **5.3** | **60.8** | **66.9** | **62.6** | **74.2** | **70.1** |
| **95%CI** | **55.6-97.1** | **5.6-81.6** | **12.2-100** | **62.5-87.2** | **1.4-11.0** | **33.4-85.1** | **42.5-87.4** | **37.6-84.6** | **58.9-87.1** | **62.0-77.6** |
| **India** | **68.9** | **6.1** | **/** | **35.3** | **/** | **12.2** | **95.2** | **/** | **/** | **43.3** |
| **95%CI** | **47.4-87.2** | **0.0-32.5** | **/** | **10.8-63.8** | **/** | **0.0-41.1** | **90.2-98.8** | **/** | **/** | **28.6-58.6** |
| **Guatemala** | **30.7** | **10.1** | **71.3** | **24.5** | **/** | **13.4** | **23.1** | **0.0** | **39.3** | **21.7** |
| **95%CI** | **8.5-57.9** | **0.0-38.8** | **31.6-98.3** | **15.1-35.0** | **/** | **0.0-49.7** | **5.5-46.3** | **0.0-3.1** | **19.5-60.8** | **14.9-29.3** |
| **China** | **27.7** | **/** | **70.8** | **17.1** | **/** | **3.2** | **8.5** | **25.5** | **10.9** | **16.8** |
| **95%CI** | **5.8-57.4** | **/** | **62.3-78.7** | **8.9-27.2** | **/** | **0.5-7.4** | **2.5-17.1** | **3.3-58.2** | **4.2-19.9** | **12.0-22.2** |
| **Ethiopia** | **18.5** | **/** | **/** | **45.9** | **/** | **0.0** | **36.4** | **13.9** | **50.9** | **33.1** |
| **95%CI** | **0.0-57.9** | **/** | **/** | **29.4-62.9** | **/** | **0.0-3.1** | **3.9-77.7** | **0.0-49.2** | **37.4-64.4** | **20.8-46.7** |
| **Benin** | **64.9** | **/** | **/** | **50.5** | **/** | **/** | **23.3** | **9.0** | **/** | **45.0** |
| **95%CI** | **60.0-69.6** | **/** | **/** | **23.6-77.2** | **/** | **/** | **19.2-27.7** | **5.3-14.0** | **/** | **26.3-64.4** |
| **Burkina Faso** | **60.6** | **/** | **/** | **66.6** | **/** | **/** | **35.6** | **3.1** | **/** | **49.7** |
| **95%CI** | **58.0-63.2** | **/** | **/** | **34.3-92.0** | **/** | **/** | **33.1-38.1** | **1.9-4.6** | **/** | **25.9-73.6** |
| **Democratic Republic of the Congo** | **39.0** | **/** | **/** | **28.6** | **/** | **/** | **9.5** | **4.0** | **/** | **22.8** |
| **95%CI** | **37.3-40.7** | **/** | **/** | **14.4-45.5** | **/** | **/** | **8.5-10.5** | **3.1-5.1** | **/** | **12.5-35.2** |
| **Mauritania** | **43.9** | **/** | **/** | **34.6** | **/** | **/** | **21.8** | **3.9** | **/** | **28.0** |
| **95%CI** | **39.4-48.5** | **/** | **/** | **15.1-57.3** | **/** | **/** | **18.1-25.7** | **1.8-7.2** | **/** | **15.0-43.2** |
| **Sierra Leone** | **78.7** | **/** | **/** | **39.1** | **/** | **/** | **32.4** | **21.7** | **/** | **47.6** |
| **95%CI** | **72.8-84.0** | **/** | **/** | **12.3-69.9** | **/** | **/** | **26.3-38.9** | **14.3-30.8** | **/** | **26.8-68.8** |
| **Togo** | **79.3** | **/** | **/** | **61.4** | **/** | **/** | **29.9** | **18.8** | **/** | **54.1** |
| **95%CI** | **73.4-84.6** | **/** | **/** | **37.2-83.0** | **/** | **/** | **23.7-36.4** | **11.7-27.8** | **/** | **35.4-72.4** |
| **Uganda** | **74.2** | **/** | **/** | **36.7** | **/** | **/** | **13.9** | **8.1** | **/** | **38.2** |
| **95%CI** | **69.8-78.3** | **/** | **/** | **29.4-44.3** | **/** | **/** | **10.7-17.4** | **4.8-12.7** | **/** | **22.9-54.7** |
| **Zimbabwe** | **94.0** | **/** | **/** | **33.1** | **/** | **/** | **26.4** | **2.2** | **/** | **46.8** |
| **95%CI** | **91.9-95.9** | **/** | **/** | **2.0-77.8** | **/** | **/** | **22.8-30.2** | **0.8-4.7** | **/** | **17.4-77.4** |
| **Type of medical institutions** | | | | | | | | | | |
| **Private** | **46.5** | **14.6** | **72.8** | **39.2** | **5.3** | **38.2** | **31.2** | **30.1** | **53.0** | **37.6** |
| **95%CI** | **25.5-68.1** | **0.0-53.6** | **12.5-100.0** | **29.1-49.8** | **1.4-11.0** | **12.9-67.3** | **16.0-48.6** | **9.1-56.4** | **33.2-72.4** | **31.0-44.4** |
| **Public** | **48.4** | **25.0** | **53.1** | **33.0** | **/** | **0.9** | **33.1** | **31.6** | **29.2** | **31.9** |
| **95%CI** | **30.7-66.3** | **8.3-45.7** | **43.1-62.9** | **22.7-44.0** | **/** | **0.0-9.6** | **15.2-53.6** | **8.9-59.5** | **14.6-46.1** | **26.0-38.0** |
| **Level of medical institutions** | | | | | | | | | | |
| **Hospital** | **57.8** | **13.9** | **53.1** | **33.2** | **/** | **3.1** | **25.9** | **35.3** | **26.5** | **32.2** |
| **95%CI** | **27.0-86.0** | **3.6-27.8** | **43.1-62.9** | **20.3-47.2** | **/** | **0.0-28.2** | **8.9-47.1** | **6.7-70.6** | **11.1-45.2** | **24.7-40.0** |
| **Primary care** | **/** | **/** | **/** | **/** | **/** | **/** | **/** | **/** | **/** | **21.7** |
| **95%CI** | **/** | **/** | **/** | **/** | **/** | **/** | **/** | **/** | **/** | **1.0-58.4** |
| **Original/Generic medicines** | | | | | | | | | | |
| **Original** | **10.6** | **0.0** | **0.0** | **18.9** | **0.0** | **54.2** | **25.1** | **58.0** | **25.9** | **20.2** |
| **95%CI** | **0.0-59.3** | **0.0-7.4** | **0.0-7.4** | **5.2-38.0** | **0.0-7.4** | **39.2-68.6** | **3.0-57.9** | **29.4-84.1** | **7.6-49.8** | **11.9-30.0** |
| **generic** | **54.7** | **72.9** | **84.9** | **37.8** | **16.7** | **20.0** | **17.7** | **15.6** | **36.8** | **34.9** |
| **95%CI** | **22.8-84.7** | **58.2-84.7** | **56.2-99.8** | **23.6-53.0** | **7.5-30.2** | **0.0-72.0** | **5.8-33.9** | **0.0-53.0** | **12.4-65.3** | **27.0-43.1** |

**Supplementary Table 5 The original data availability of global children essential medicines in 2016-2020**

| **ATC** | **A** | **B** | **C** | **D** | **H** | **J** | **L** | **M** | **N** | **P** | **R** | **V** | **Overall** |
| --- | --- | --- | --- | --- | --- | --- | --- | --- | --- | --- | --- | --- | --- |
| **Global** | **43.5** | **33.2** | **31.9** | **19.9** | **30.3** | **27.6** | **69.9** | **26.1** | **21.6** | **35.8** | **26.2** | **87.1** | **43.1** |
| **95%CI** | **31.3-56.1** | **14.6-54.8** | **17.9-47.6** | **10.3-31.5** | **15.9-46.5** | **22.2-33.3** | **64.7-74.9** | **13.8-40.2** | **13.1-31.4** | **15.6-58.9** | **16.8-36.7** | **78.2-94.3** | **40.1-46.2** |
| **World bank county-income groups** | | | | | | | | | | | | | |
| **LIC** | **49.4** | **50.6** | **/** | **/** | **/** | **34.1** | **93.7** | **47.7** | **24.9** | **26.6** | **19.2** | **95.6** | **48** |
| **95%CI** | **14.3-84.8** | **6.6-94.0** | **/** | **/** | **/** | **21.2-48.3** | **88.5-97.8** | **0.0-99.3** | **9.9-43.6** | **1.9-63.8** | **0.0-62.1** | **75.5-100.0** | **38.8-57.2** |
| **LMC** | **85.0** | **70.7** | **44.9** | **/** | **48.2** | **47.7** | **50.4** | **30.0** | **48.0** | **56.0** | **38.1** | **69.4** | **51.3** |
| **95%CI** | **63.6-99.0** | **45.5-91.2** | **20.6-70.4** | **/** | **21.4-75.4** | **32.3-63.3** | **43.2-57.5** | **9.0-55.1** | **17.8-79.1** | **25.8-84.0** | **11.0-69.9** | **44.7-90.1** | **45.6-57.1** |
| **UMC** | **23.1** | **17.1** | **19.7** | **19.9** | **15.1** | **18.2** | **67.6** | **18.6** | **12.6** | **14.5** | **26.5** | **76.7** | **24.4** |
| **95%CI** | **13.9-33.6** | **3.3-37.4** | **6.0-38.2** | **10.3-31.5** | **4.2-30.2** | **13.6-23.2** | **56.0-78.2** | **8.0-32.0** | **3.3-37.4** | **6.5-26.7** | **16.7-37.5** | **56.7-92.3** | **21.5-27.4** |
| **HIC** | **91.9** | **/** | **/** | **/** | **/** | **/** | **95.7** | **/** | **/** | **/** | **/** | **97.3** | **95.8** |
| **95%CI** | **72.4-100.0** | **/** | **/** | **/** | **/** | **/** | **93.2-97.8** | **/** | **/** | **/** | **/** | **90.4-100.0** | **93.5-97.7** |
| **Country** | | | | | | | | | | | | | |
| **China** | **21.8** | **17.1** | **19.7** | **19.9** | **15.1** | **18.2** | **26.1** | **18.6** | **12.6** | **14.5** | **26.5** | **/** | **18.8** |
| **95%CI** | **12.8-32.3** | **3.3-37.4** | **6.0-38.2** | **10.3-31.5** | **4.2-30.2** | **13.6-23.2** | **13.4-41.1** | **8.0-32.0** | **6.3-20.6** | **6.5-26.7** | **16.7-37.5** | **/** | **16.2-21.5** |
| **Ethiopia** | **42.7** | **50.6** | **/** | **/** | **/** | **34.1** | **/** | **47.7** | **24.9** | **26.6** | **19.2** | **/** | **32.1** |
| **95%CI** | **8.9-100.0** | **6.6-94.0** | **/** | **/** | **/** | **21.2-48.3** | **/** | **0.0-99.3** | **9.9-43.6** | **1.9-63.8** | **0.0-62.1** | **/** | **23.1-41.9** |
| **Ghana** | **98.0** | **/** | **/** | **/** | **52.8** | **/** | **55.9** | **67.7** | **/** | **/** | **/** | **28.0** | **58.7** |
| **95%CI** | **73.1-100.0** | **/** | **/** | **/** | **16.7-87.7** | **/** | **37.1-74.1** | **33.5-95.2** | **/** | **/** | **/** | **0.0-75.5** | **44.2-72.6** |
| **India** | **98.4** | **/** | **/** | **/** | **46.3** | **75.7** | **39.7** | **/** | **/** | **/** | **/** | **/** | **45.9** |
| **95%CI** | **84.0-100.0** | **/** | **/** | **/** | **13.6-80.4** | **39.6-99.5** | **31.5-48.2** | **/** | **/** | **/** | **/** | **/** | **36.9-55.0** |
| **Mongolia** | **/** | **/** | **/** | **/** | **/** | **/** | **/** | **/** | **/** | **/** | **/** | **/** | **69.2** |
| **95%CI** | **/** | **/** | **/** | **/** | **/** | **/** | **/** | **/** | **/** | **/** | **/** | **/** | **58.6-79.0** |
| **Nigeria** | **/** | **70.7** | **44.9** | **/** | **/** | **/** | **/** | **/** | **/** | **/** | **/** | **/** | **45.9** |
| **95%CI** | **/** | **45.4-91.2** | **20.6-70.4** | **/** | **/** | **/** | **/** | **/** | **/** | **/** | **/** | **/** | **36.9-55.0** |
| **Vietnam** | **60.7** | **/** | **/** | **/** | **/** | **42.9** | **/** | **17.7** | **48.0** | **56.0** | **38.1** | **/** | **42.2** |
| **95%CI** | **24.9-91.1** | **/** | **/** | **/** | **/** | **27.4-59.2** | **/** | **3.1-40.1** | **17.8-79.1** | **25.8-84.0** | **11.0-69.9** | **/** | **31.1-53.7** |
| **Type of medical institutions** | | | | | | | | | | | | | |
| **Private** | **42.8** | **27.4** | **9.3** | **28.8** | **38.7** | **22.2** | **49.0** | **28.6** | **16.1** | **34.6** | **25.7** | **50.0** | **32.0** |
| **95%CI** | **22.7-64.0** | **0.0-73.0** | **0.0-35.2** | **11.2-50.2** | **15.7-64.1** | **14.3-31.2** | **39.5-58.6** | **10.8-50.1** | **6.4-28.6** | **10.4-63.7** | **9.5-46.1** | **6.8-93.2** | **27.0-37.1** |
| **Public** | **37.1** | **26.8** | **28.6** | **15.3** | **22.0** | **33.0** | **28.7** | **21.9** | **30.5** | **53.7** | **24.9** | **0.0** | **29.4** |
| **95%CI** | **21.9-53.4** | **6.9-53.1** | **5.4-59.8** | **5.4-28.7** | **5.7-42.7** | **25.8-40.6** | **19.4-38.6** | **4.5-45.2** | **15.7-47.6** | **40.8-66.4** | **14.3-37.1** | **0.0-84.2** | **25.3-33.6** |
| **Level of medical institutions** | | | | | | | | | | | | | |
| **Hospital** | **24.5** | **25.2** | **14.0** | **12.2** | **39.0** | **27.5** | **42.6** | **39.4** | **14.9** | **8.4** | **34.4** | **0.0** | **29.9** |
| **95%CI** | **12.5-38.3** | **17.4-33.8** | **0.4-37.7** | **0.9-32.0** | **16.0-64.0** | **20.2-35.4** | **33.1-52.3** | **24.3-55.3** | **2.5-33.3** | **0.0-27.4** | **19.0-51.5** | **0.0-84.2** | **25.6-34.3** |
| **Primary care** | **71.4** | **66.7** | **50.0** | **/** | **/** | **59.1** | **/** | **73.7** | **28.7** | **50.9** | **37.3** | **/** | **47.7** |
| **95%CI** | **45.3-92.1** | **47.2-82.7** | **22.6-77.4** | **/** | **/** | **44.5-73.1** | **/** | **57.3-87.7** | **7.1-56.4** | **17.6-83.8** | **0.0-95.6** | **/** | **37.3-58.1** |
| **Original/Generic medicines** | | | | | | | | | | | | | |
| **Original** | **7.2** | **2.5** | **0.1** | **22.0** | **4.6** | **7.7** | **26.6** | **12.9** | **8.0** | **/** | **17.3** | **/** | **9.2** |
| **95%CI** | **0.6-17.8** | **0.0-13.8** | **0.0-1.9** | **7.3-41.3** | **0.0-24.7** | **3.6-13.0** | **9.5-48.1** | **1.5-31.2** | **1.1-19.2** | **/** | **5.2-33.9** | **/** | **6.4-12.2** |
| **Generic** | **47.4** | **40.0** | **47.5** | **17.9** | **37.1** | **28.7** | **47.8** | **28.5** | **18.5** | **/** | **33.8** | **28.0** | **32.2** |
| **95%CI** | **33.9-61.1** | **31.0-49.2** | **23.5-72.0** | **6.4-33.0** | **19.4-56.2** | **21.8-36.0** | **32.1-63.8** | **9.3-51.7** | **7.3-32.9** | **/** | **20.6-48.3** | **0.0-75.5** | **28.1-36.5** |

*A, Alimentary Tract And Metabolism; B, Blood And Blood Forming Organs; C, Cardiovascular System; D, Dermatologicals; G, Genito Urinary System And Sex Hormones; H, Systemic Hormonal Preparations, Excl. Sex Hormones And insulin; J, Antiinfectives For Systemic Use; L, Antineoplastic And Immunomodulating Agents; M, Musculo-Skeletal System; N, Nervous System; P, Antiparasitic Products, Insecticides And Repellents; R, Respiratory System; S, Sensory Organs*

**Supplementary Table 6. Characteristics of excluded studies**

| Number | Study | Reason for exclusion |
| --- | --- | --- |
| 1 | Ridde, 2005 | The full text is not available. |
| 2 | Ranganathan, 2010 | The full text is not available. |
| 3 | Auste, 2012 | The full text is not available. |
| 4 | Rane, 2014 | The full text is not available. |
| 5 | Kazaryan, 2015 | The full text is not available. |
| 6 | Hailu, 2016 | The full text is not available. |
| 7 | Brooke, 2017 | The full text is not available. |
| 8 | Chowdhury, 2018 | The full text is not available. |
| 9 | Boateng, 2020 | The full text is not available. |
| 10 | Faruqui, 2018 | The full text is not available. |
| 11 | Kazaryan, 2018 | The full text is not available. |
| 12 | Gunasekera, 2020 | The full text is not available. |
| 13 | WHO, 2006 | No related data could be extracted. |
| 14 | Kamuhabwa, 2016 | No related data could be extracted. |
| 15 | Chalker, 1995 | No related data could be extracted. |
| 16 | Zucker, 2007 | No related data could be extracted. |
| 17 | Robertson, 2009 | No related data could be extracted. |
| 18 | Shafiq, 2011 | No related data could be extracted. |
| 19 | Chandani, 2012 | No related data could be extracted. |
| 20 | Li, 2012 | No related data could be extracted. |
| 21 | Sun, 2012 | No related data could be extracted. |
| 22 | Balasubramaniam, 2014 | No related data could be extracted. |
| 23 | Li, 2014 | No related data could be extracted. |
| 24 | Shi, 2014 | No related data could be extracted. |
| 25 | Babigumira, 2017 | No related data could be extracted. |
| 26 | Božić, 2017 | No related data could be extracted. |
| 27 | Orubu, 2017 | No related data could be extracted. |
| 28 | Perumal-Pillay, 2017 | No related data could be extracted. |
| 29 | Cohen, 2018 | No related data could be extracted. |
| 30 | Boateng, 2019 | No related data could be extracted. |
| 31 | Burns, 2019 | No related data could be extracted. |
| 32 | Simonyan, 2019 | No related data could be extracted. |
| 33 | Boateng, 2020 | No related data could be extracted. |
| 34 | delMoral-Sanchez, 2020 | No related data could be extracted. |
| 35 | Hailu, 2020 | No related data could be extracted. |
| 36 | Roy, 2021 | No related data could be extracted. |
| 37 | Sri Ranganathan, 2021 | No related data could be extracted. |
| 38 | Vassal,2021 | No related data could be extracted. |
| 39 | Dai, 2020 | Duplicated with Chinese edition. |
| 40 | Wang, 2014 | Duplicated with English edition. |
| 41 | Ravindran, 2012 | The availability rate is defined as the percentage of surveyed medicines in the WHO 20 core medicines, which is different from our included criteria. |
| 42 | Yang, 2012 | The availability rate is defined as the percentage of countries globally and by WHO region that included each priority medicine on its national medicines list, which is different from our included criteria. |
| 43 | Nascimento, 2017 | Not related to children. |
| 44 | Mensah, 2020 | Not related to children. |
| 45 | Liu, 2015 | Not related to availability. |
| 46 | Kabunga, 2017 | Not related to availability. |
| 47 | Unguru, 2019 | Not related to availability. |
| 48 | Abu-Arja, 2021 | Not related to availability. |
